# Switchable biomimetic nanochannels for on-demand SO_2_ detection by light-controlled photochromism

**DOI:** 10.1038/s41467-023-37654-y

**Published:** 2023-04-05

**Authors:** Dan Zhang, Yongjie Sun, Zhichao Wang, Fang Liu, Xuanjun Zhang

**Affiliations:** 1 Faculty of Health Sciences, University of Macau, Taipa, Macau SAR, 999078 China; 2 MOE Frontiers Science Center for Precision Oncology, University of Macau, Taipa, Macau SAR, 999078 China

**Keywords:** Sensors, Nanopores, Nanofluidics, Surface assembly, Sensors and biosensors

## Abstract

In contrast to the conventional passive reaction to analytes, here, we create a proof-of-concept nanochannel system capable of on-demand recognition of the target to achieve an unbiased response. Inspired by light-activatable biological channelrhodopsin-2, photochromic spiropyran/anodic aluminium oxide nanochannel sensors are constructed to realize a light-controlled inert/active-switchable response to SO_2_ by ionic transport behaviour. We find that light can finely regulate the reactivity of the nanochannels for the on-demand detection of SO_2_. Pristine spiropyran/anodic aluminium oxide nanochannels are not reactive to SO_2_. After ultraviolet irradiation of the nanochannels, spiropyran isomerizes to merocyanine with a carbon‒carbon double bond nucleophilic site, which can react with SO_2_ to generate a new hydrophilic adduct. Benefiting from increasing asymmetric wettability, the proposed device exhibits a robust photoactivated detection performance in SO_2_ detection in the range from 10 nM to 1 mM achieved by monitoring the rectified current.

## Abstract (web summary)

Conventional nanochannel sensors are passively responsive and may be slowly damaged by analytes present in the environment before detection. Here, authors developed a light-controlled inert/active-switchable biomimetic nanochannel sensor to achieve SO_2_ on-demand detection and long-term preservation.

## Introduction

The diversification of nanofabrication and modification techniques has led to the development of efficient and delicate artificial nanochannel sensors^1–7^. However, while there are various strategies for producing nanochannels with structures comparable to those of their natural counterparts^8^, replicating their precise sensing and transport behaviour, such as controlled switching and on-demand transport, remains challenging^9,10^. In conventional sensing systems, the probes immobilized on nanochannels are always reactive. Due to the large specific surface area of the nanochannel, the probes can easily interact with other substances, either by exposure to air or by immersion in solution^2,11^. For example, probe molecules have already partially reacted with the analytes present in the natural environment before the intended samples are detected. This passive response mode^12^ inevitably results in background signals, reduced detection precision, or even damage to the nanochannel sensor. To achieve the level of precision sensing found in nature, it is necessary to develop a tuneable nanochannel sensor that can satisfy the requirements of real-time, on-demand detection.

**Fig. 1:**
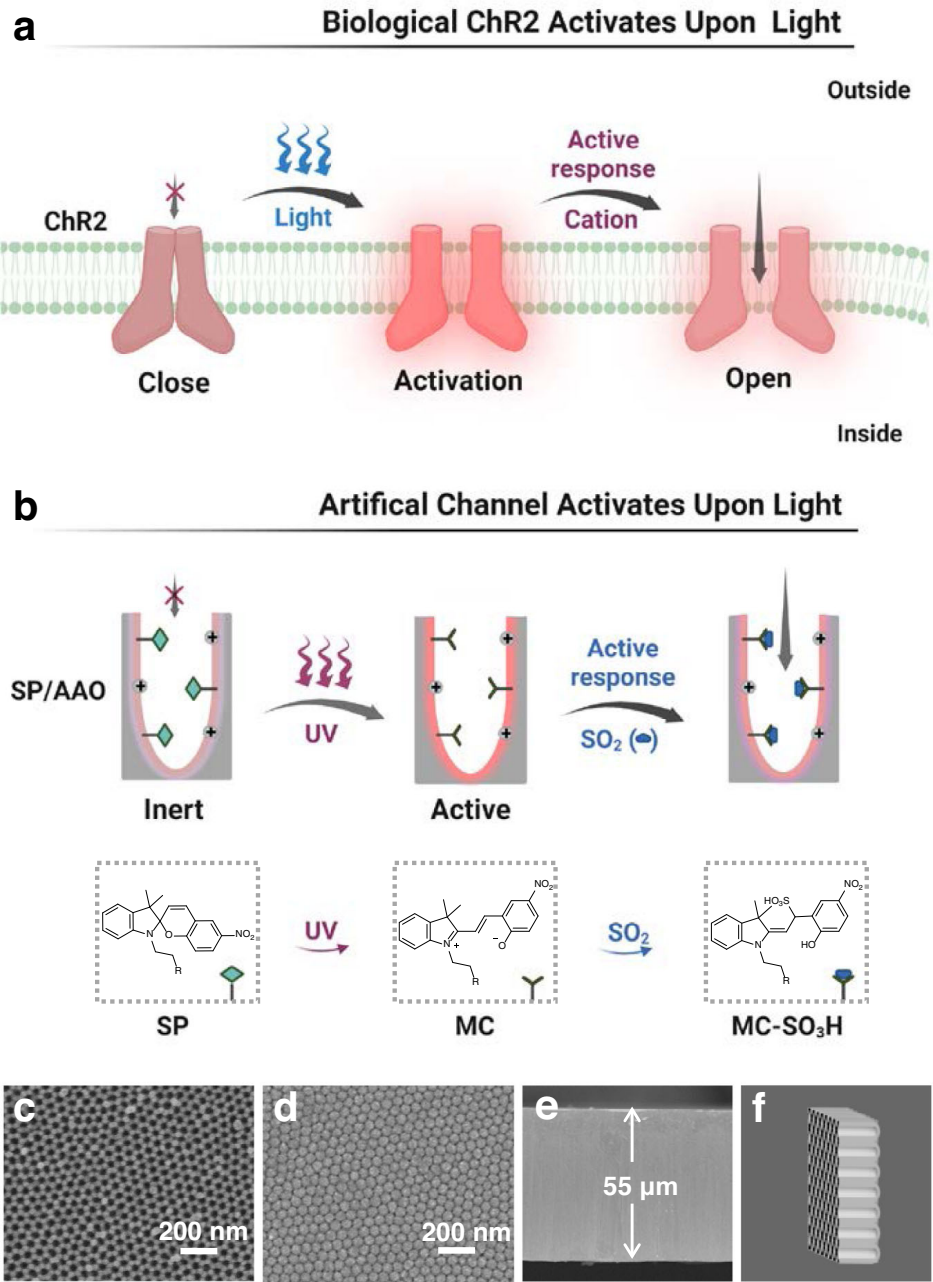
Schematic illustration of the ChR2-inspired light-controlled nanochannel sensor with inert/active-switchable reactivity for on-demand detection of SO_2_ and the chemical structures of the probes. **a** Biological ChR2 activation upon light exposure. **b** Biomimetic SP/AAO nanochannels with UV-controlled inert/active-switchable response to SO_2_. Figure 1a, b was created with BioRender.com. SEM images of (**c**) the top porous side, (**d**) the bottom blocking layer, and (**e**) the cross-section of the porous tubular AAO nanochannels. **f** Schematic diagram of the tubular AAO nanochannels. ChR2 channelrhodopsin-2, SP spiropyran, MC merocyanine, MC–SO_3_H merocyanine and SO_2_ adducts. UV ultraviolet, SP/AAO spiropyran/anodic aluminium oxide.

Herein, we envision that light can be creatively utilized to elicit nanochannel reactivity prior to analyte binding, which allows for the construction of light-controlled inert/active state-switchable recognizers to achieve an unbiased real-time response. This is similar to the light-sensitive channelrhodopsin-2 (ChR2) protein channels found in nature^13,14^. When irradiated with blue light, the closed ChR2 conformation changes and is activated, allowing inward cation flow (Fig. 1a). Mimicking nature, biomimetic light-governed nanochannels are attractive due to their advantages of noninvasive stimulus and remote spatiotemporal control^15–19^. In particular, photochromic molecules possess the unique ability to perform reversible interconversion between isomeric bistables by alternate irradiation with ultraviolet (UV) and visible (Vis) light^20–22^. In addition to this structural transformation, the reactivity of the photochromic molecules may also be reversibly modulated by the corresponding light stimulus because of the rearrangement of their electronic configurations^12,23^. It has been reported that the structural transformation of photochromic probes in nanochannel systems is highly effective in constructing ion conduction switches via a light-controlled approach^24,25^. However, the potential role of this structural transformation in activating nanochannel reactivity to analytes has not been systematically studied. Light can convert photochromic probes on the inner surface of channels from one isomer that is inert to the analyte to another that is reactive to the analyte, which opens up the possibility of creating a nanosensor with a light-governed, on-demand response to a specific analyte. In addition, the photochromic action and the corresponding response behaviour can be visualized by colour change, providing a convenient way to rule out false-positive/negative signals^26,27^.

As a novel signalling molecule, endogenous sulfur dioxide (SO_2_) plays a key role in cardiovascular regulation in living organisms^28^. In addition, SO_2_ is a common toxic pollutant. Excessive intake of SO_2_ has been suggested to contribute to cardiovascular diseases or age-related disorders^29,30^. In recent years, SO_2_ has been allowed to be used more frequently in the alcoholic beverage, food, and pharmaceutical industries as a bacteriostatic, preservative, and antioxidant agent^31^. Therefore, it is highly desirable to develop high-performance sensors for the accurate determination of SO_2_ and realize their application in various sample essays. The number of solution-based fluorescent probes for SO_2_ is quickly increasing^12,32,33^, but the development of nanochannel-based SO_2_ detectors is still in the early stage. In 2020, Sun et al. reported the first and only SO_2_ nanochannel sensor^34^, which was constructed by modifying the nanochannel surface with active ketone groups. Relying on the passive nucleophilic addition of ketone probes and SO_2_, this nanofluidic device was capable of detecting SO_2_ down to μM level.

In this work, we developed an inert/active state-switchable sensor and used the detection of SO_2_ as an example to investigate the light-controlled on-demand recognition behaviour of a spiropyran (SP)/anodic aluminium oxide (AAO) nanochannel system (Fig. 1b). SP is a typical photochromic molecule, and its photoisomerization behaviour has been well demonstrated^23–27,35–37^. Upon UV irradiation, it can isomerize to merocyanine (MC), leading to distinct changes in structure and reactivity^12,23^. The UV-activated MC form has an unsaturated carbon‒carbon double bond (C ═ C) that can act as a nucleophilic site for SO_2_ attack (Fig. 1b)^38^. Therefore, the SP/AAO nanochannels were capable of on-demand activation when applied for detection, which indicated their potential as superior candidates for the design of light-driven artificial nanosensors to actively recognize SO_2_. Especially for the high levels of SO_2_ in the environment, such photomodulated on-demand responses could subtly avoid undesired interactions between nanochannels and ambient SO_2_. This study demonstrated biomimetic nanochannels with a light-governed inert/active-switchable response functionality and provided an exciting approach for creating on-demand and visual nanofluidic detectors in a precise light-controlled manner.

## Results

### Asymmetric immobilization of SP on AAO nanochannels

**Fig. 2:**
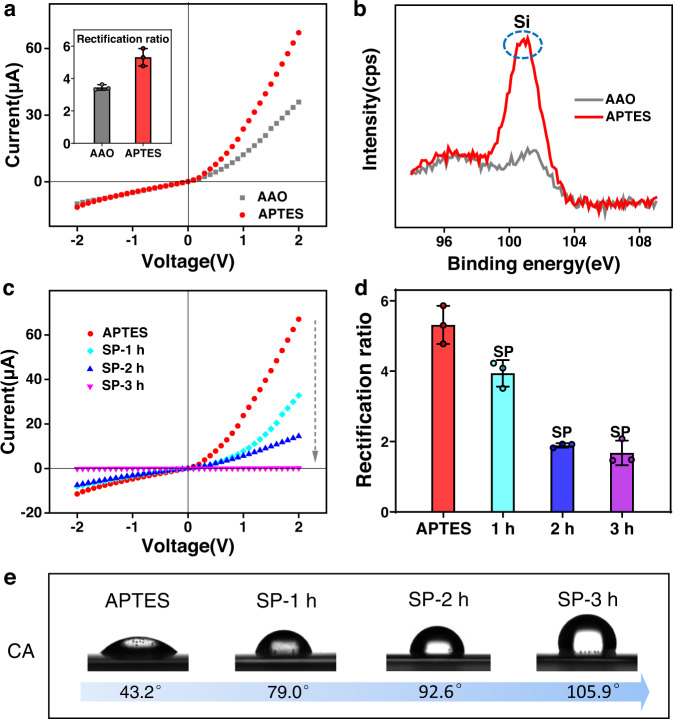
Fabrication and characterization of SP/AAO nanochannels. **a** I–V curves and (**b**) XPS features of the AAO nanochannels before and after APTES treatment. **c** I–V curves, (**d**) rectification ratios, and (**e**) CA measurements of APTES-treated nanochannels before and after modification with SP. Error bars in the inset of a and in d denote the standard deviation from three different samples. AAO anodic aluminium oxide. APTES: (3-aminopropyl)triethoxysilane. cps counts per second. SP-1 h SP/AAO nanochannels obtained by spiropyran modification for 1 h. SP-2 h SP/AAO nanochannels obtained by spiropyran modification for 2 h. SP-3 h SP/AAO nanochannels obtained by spiropyran modification for 3 h. CA contact angle.

As shown in Fig. 1b, the artificial AAO nanochannels were modified with SP and functioned as a light-regulated inert/active-state switchable detector for on-demand response to SO_2_. Typical scanning electron microscopy (SEM) observations showed that the top surface of the original AAO had an opened porous structure with a pore diameter of approximately 25 nm (Fig. 1c). However, the bottom surface of the AAO nanochannels was sealed by hexagonal close-packed arrays of hemispherical domes, forming a critical nanoconfinement region (Fig. 1d)^15^. Parallel channels existed between the top and bottom surfaces with a thickness of approximately 55 μm (Fig. 1e). As a result, tubular nanochannels with maximum geometric asymmetry were formed (Fig. 1f), which enabled the AAO membrane to realize optimal ion transport properties, including ion rectification and ion selectivity. As shown in Fig. 2a, the tubular AAO nanochannels could rectify ionic currents due to the plentiful protonated hydroxyl groups (OH_2_^+^) on their inner surface^39,40^. Additionally, the AAO nanochannels could work as an asymmetric rigid scaffold for subsequent covalent functionalization (Supplementary Fig. 1). The AAO membrane was first aminated by immersion in (3-aminopropyl)triethoxysilane (APTES) solution. Upon completion of the treatment, a clear increase in ionic current from approximately 35.9 to 67.1 μA at 2 V could be observed (Fig. 2a), which mainly originated from the increase in the positive amino charges on the APTES surface, leading to enhanced rectification performance (Fig. 2a inset)^41^. The rectification ratio was defined as the ratio of the absolute values of the ionic current at +2 V to those at −2 V. The emergence of a new silicon peak at approximately 101 eV as evidenced by X-ray photoelectron spectroscopy (XPS) further confirmed the successful attachment of APTES on the AAO nanochannels (Fig. 2b)^24,42^. Having immobilized APTES, carboxy-functionalized SP probes (Supplementary Figs. 2–5) were chemically patterned on the porous side of the nanochannels, which was implemented through a diffusion-limited patterning (DLP) method (Supplementary Fig. 6)^24,43^. The SEM results showed that the modified AAO remained intact, suggesting that this method was quite mild (Supplementary Fig. 7). In addition, the successful modification of SP could be proven by peak fitting of the XPS spectrum results (Supplementary Fig. 8). These peaks found at approximately 399.9 eV and 288.4 eV (corresponding to the nitrogen and carbon atoms of the amide units) allowed us to clearly demonstrate the covalent linkages of the SP probes on the APTES-modified AAO channel via 1-ethyl-3-(3-dimethylaminopropyl) carbodiimide hydrochloride/N-hydroxysuccinimide (EDC/NHS) chemistry^44^.

By measuring the ionic transport and surface wettability, we further investigated the SP molecule-patterned nanochannels at different modification times (Fig. 2c–e). As demonstrated in the current–voltage (I–V) curves, the ionic current at 2 V gradually decreased with the modification time (Fig. 2c). Time-dependent contact angle (CA) measurements revealed that the top surface of the nanochannel membrane became increasingly hydrophobic due to the increase in the number of neutral SP molecules (Fig. 2e)^45,46^. After the initial 1 h of treatment with SP, the ionic current decreased from approximately 67.1 to 32.8 μA at +2 V and from approximately −11.4 to −8.04 μA at −2 V. As a consequence, the nanochannel rectification ratio decreased slightly (Fig. 2d). This indicated that the density of available SP was still not high, which may have weakened the light response effect and detection sensitivity of the system. However, after 2 h, both the ion current at 2 V and the rectification ratio significantly decreased. This phenomenon suggested that an appropriate amount of SP was attached to the APTES surface, resulting in reduced membrane wettability and surface charges. When the treatment was continued for 3 h, the current decreased to 10^−7^ A. The immobilization of excess SP apparently lowered the surface wettability of the nanochannels, leading to a highly hydrophobic interface on the top side with a CA of ≈105.9°. This severely hindered the smooth ion transport across the membrane. A time-dependent increase in the amount of SP was further evidenced by the reduced zeta potential and ion rectification ratio (Supplementary Fig. 9 and Fig. 2d). The positive charges on the amino groups of APTES and hydroxyl groups of AAO were gradually covered by neutral SP, resulting in a decrease in the positive charge density on the inner surface of the nanochannels. However, no obvious change in CA was observed from the bottom surface (Supplementary Fig. 10), suggesting that SP could be finely patterned on the porous side of the tubular nanochannels to build asymmetrical SP/AAO nanochannels. The detailed statistical results of the ion rectification ratio showed a low standard deviation (SD, Supplementary Table 1), intuitively indicating its high reproducibility. The comparison of three different modification times demonstrated that the optimal modification states and ion transport properties could be achieved at 2 h; thus, the SP/AAO system with 2 h of treatment was further applied in SO_2_ sensing.

### Inert/active-switchable nanosensor for on-demand response to SO_2_

**Fig. 3:**
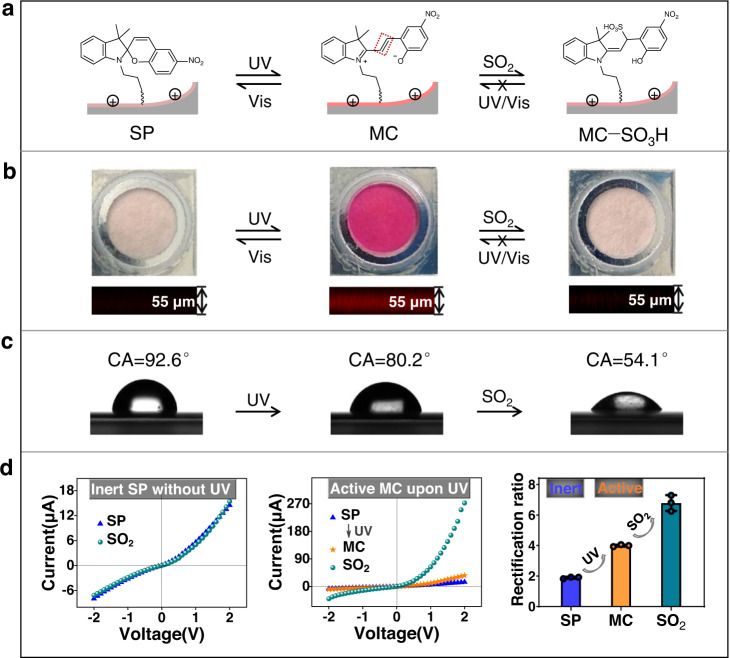
Light-controlled inert/active state-switchable sensor for SO_2_ and the photochromic properties of the SP/AAO nanochannels. **a** Schematic diagram of the light-controlled SP/AAO nanochannel sensor for SO_2_. The red dashed box is the C = C response site in MC form. **b** Change in optical colour and confocal fluorescence and (**c**) CA measurements of SP/AAO nanochannels during the light-controlled detection of SO_2_. **d** I–V curves of SP/AAO nanochannels treated with SO_2_ (left panel) before and (middle panel) after UV radiation, as well as (right panel) the rectification ratio changes of SP/AAO nanochannels. Error bars in d denote the standard deviation from three different samples. SP spiropyran, MC merocyanine, MC–SO_3_H merocyanine and SO_2_ adducts, UV ultraviolet, Vis visible, CA contact angle.

The on-demand recognition of SO_2_ in the nanochannels was realized based on the light-elicited isomerization of inert SP to activated MC, generating unsaturated C ═ C, which is capable of binding to SO_2_ through nucleophilic addition to yield a new MC−SO_3_H adduct (Fig. 3a). Benefiting from the photochromic properties of spiropyran, the light-controlled response process of the nanochannel systems could be tracked distinctly by visual optical colour and laser scanning confocal microscopy (LSCM) (Fig. 3b). Initially, the SP molecule was predominantly in the closed spirocyclic state and structurally unconjugated^47^. As a result, the SP-modified nanochannel membrane showed an optically colourless and weak fluorescence. Under UV light irradiation, however, the C−O bond of SP was cleaved and isomerized to a ring-opened MC form with a C ═ C linkage. At this point, the entire molecule formed an extended large conjugation plane^48,49^, resulting in the purple colour of the top surface and the intense red fluorescence of the cross-section, indicative of an MC-state nanochannel membrane. In addition, these optical results also spatially demonstrated that spiropyrane probes were uniformly grafted to the nanochannel surface. By comparison, no colour change occurred for the SP/AAO nanochannels after 10 min of exposure to other bands of light sources, such as visible and infrared light (Supplementary Fig. 11). This implied that, unlike UV, these longer wavelengths of light were unable to convert SP into MC. Additionally, the shorter wavelength X-ray sources for XPS could not isomerize SP to its MC form because no characteristic N^+^ peak of ring-opening MC was observed (Supplementary Fig. 8)^50^. These results suggested that UV irradiation played a pivotal role in the photoisomerization conversion of SP. When the UV light was off and the visible light was on, the optical colour and fluorescence of the membrane reversibly returned to their original states as a result of reverse photochemical isomerization from MC to SP. The photochromism process of SP/AAO nanochannels showed good reversibility through alternating UV/Vis treatment, which is similar to the writing, reading, and erasing of information that occurs for SP optical storage memory^51^. These distinctive optical variations were associated with the reversible conversion of the structural and electronic reorganization of spiropyran. Most notably, the UV-activated MC had an unsaturated C ═ C bond that was reactive towards nucleophiles^12,23,52–54^. The purple colour and red fluorescence of the nanochannel membrane disappeared after SO_2_ treatment. This was due to the nucleophilic attack of SO_2_ towards C ═ C forming the MC−SO_3_H adduct, which broke the π-conjugated structure of MC^55^. The waxing and waning of the MC absorption peak centred at approximately 550 nm during UV irradiation and SO_2_ exposure also indicated the corresponding conversion process between SP, MC, and MC−SO_3_H (Supplementary Fig. 12)^54^. However, once the fluorescence and colour of the membrane faded, it was difficult to restore regardless of the alternating UV/Vis irradiation. Such a feature supported the idea that new MC−SO_3_H adducts were generated after treatment of MC-state nanochannels with SO_2_. The increase in the intensity of the S peak at 168.4 eV and the ratio of S atoms indicated by the XPS data further supported the successful reaction of SO_2_ with MC on the AAO membrane (Supplementary Fig. 13 and Supplementary Table 2)^56,57^. Our sensor was designed to be switchable before detection but ‘locked’ after detection. This not only extended the shelf life before detection and enabled an on-demand response but also prevented reversibility after detection, making the data more reliable.

Based on this proof-of-concept strategy, we first determined that SP/AAO nanochannels before UV irradiation were not reactive to SO_2_ by measuring typical I−V curves and CA changes (Fig. 3c, d). As shown in the left panel of Fig. 3d, there was no obvious difference in the ionic current when the SP-modified nanochannels were treated with SO_2_. The I–V characteristics demonstrated that SP was chemically inert and that its reactivity towards SO_2_ was inhibited before UV irradiation. When exposed to UV irradiation prior to the addition of SO_2_, we observed a slight increase in the ionic currents of the SP/AAO nanochannels, resulting in the generation of a light response current, defined as the UV response current (I_UV_). This is because upon UV irradiation, SP converted to its charge-separated zwitterionic MC state in a slightly acidic environment (pH 5.5)^12,54^, enhancing the wettability of the top interface of the nanochannel membrane (Fig. 3c)^27,46^. At this point, once the MC-state nanochannels were treated with SO_2_, the ionic current at +2 V immediately increased from ~36.1 to 273 μA, showing a satisfactory SO_2_ response current (${{{\rm{I}}}_{{{{\rm{SO}}}}_{2}}}$) of ≈237 μA (middle panel in Fig. 3d). ${{{\rm{I}}}_{{{{\rm{SO}}}}_{2}}}$ was defined as the difference between the 2 V ionic current after SO_2_ treatment and the 2 V ionic current under UV irradiation. Such a high ${{{\rm{I}}}_{{{{\rm{SO}}}}_{2}}}$ was due to the nucleophilic addition reaction between the MC component and SO_2_ forming MC−SO_3_H adducts (Supplementary Fig. 14), which resulted in a reversal of the wettability properties of the top surface of the nanochannels from hydrophobic (CA = 80.2°) to hydrophilic (CA = 54.1°) (Fig. 3c). However, there was no visible change in the hydrophobicity of the bottom surface during the light-governed recognition of SO_2_ due to the absence of reaction behaviour (Supplementary Fig. 15). Accordingly, asymmetric Janus wettability, i.e., a hydrophilic top surface and a hydrophobic bottom surface, was established on both sides of the SP/AAO nanochannels. As we recently reported^58^, the establishment of Janus wettability not only gated ion transport in the nanochannels but also allowed the nanochannels to achieve an ion rectification effect^59,60^. In the right panel of Fig. 3d, the rectification ratio (Supplementary Table 3) of the SP/AAO nanochannels progressively increased during the UV-stimulated SO_2_ response. Together, the results demonstrated that the sensor could be activated on demand through the conversion of the SP probes to their MC isomers upon UV irradiation. Mass spectrometry (MS) analysis further confirmed the reactivity of active MC, rather than inert SP, to SO_2_ (Supplementary Fig. 16). As a result, an inert/active state-switchable artificial nanofluidic sensor for SO_2_ modulated by remote UV light was successfully constructed for the first time. Such a subtle design facilitated the long-term preservation of the nanochannel sensor and real-time on-demand detection.

### Effect of photoactivation on SO_2_ response behavior

**Fig. 4:**
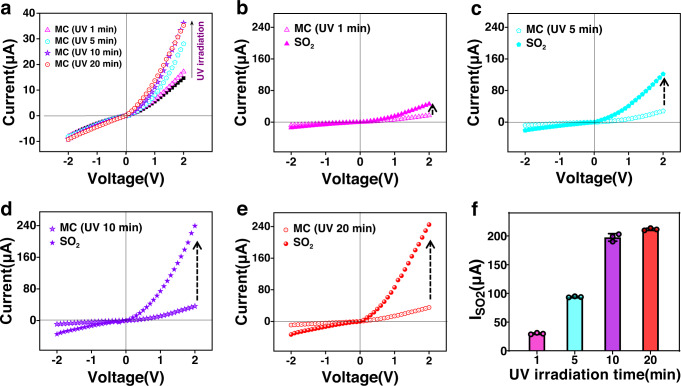
Effect of the photoactivation state on SO_2_ response. **a** I−V curves of SP/AAO nanochannels after different UV irradiation times. I−V curves of SP/AAO nanochannels treated with SO_2_ after UV activation for (**b**) 1 min, (**c**) 5 min, (**d**) 10 min, and (**e**) 20 min. **f** Corresponding SO_2_ response current (${{{\rm{I}}}_{{{{\rm{SO}}}}_{2}}}$). Error bars in f denote the standard deviation from three different samples. MC (UV 1 min): MC-state nanochannels obtained after 1 min of UV irradiation. MC (UV 5 min): MC-state nanochannels obtained after 5 min of UV irradiation. MC (UV 10 min): MC-state nanochannels obtained after 10 min of UV irradiation. MC (UV 20 min): MC-state nanochannels obtained after 20 min of UV irradiation.

To further verify the vital role of UV irradiation in the SO_2_ response, the time period of irradiation applied to the SP/AAO nanochannel sensing system was regulated. For SP/AAO channels with 2 h modification, I_UV_ increased with increasing UV irradiation time from 0 to 20 min (Supplementary Fig. 17) and showed satisfactory repeatability (Supplementary Table 4). In the dark state, the ionic current of the SP/AAO nanochannel system remained at 14.7 μA. When incrementally regulating the time period of UV irradiation applied to the SP/AAO nanochannels, the ionic current at 2 V gradually increased to ~36 μA (Fig. 4a). The photocurrent response trend proved that the remote UV stimulus progressively achieved the transition from the inert SP state to the activated MC state in the photochromic channels, ultimately attaining the highest MC yield and maximal nanochannel activity state within 10 min. As a visual indicator, the colour deepening of the channel membrane from colourless to pink to purple further demonstrated the multilevel photoconversion behaviour (Supplementary Fig. 18). Then, an identical concentration of SO_2_ (100 μM) was separately introduced into the detection system with different activation states to investigate the influence of light regulation on the SO_2_ response. When in contact with SO_2_, the ionic current in the SP/AAO nanochannel after 1 min of UV stimulus increased slightly, leading to an ${{{\rm{I}}}_{{{{\rm{SO}}}}_{2}}}$ of ≈28.8 µA (Fig. 4b). Within the first 1 min, the response rate in the current was calculated to be 0.48 μA s^−1^. For the membrane irradiated for 5 min, a distinct increase in current was recorded after SO_2_ stimulus, which resulted in an ${{{\rm{I}}}_{{{{\rm{SO}}}}_{2}}}$ production of ≈93.6 μA (Fig. 4c). After the nanosensor was irradiated for 10 min, a high ionic current of ~239.2 μA was detected, resulting in the generation of ${{{\rm{I}}}_{{{{\rm{SO}}}}_{2}}}$ as high as 203.1 μA (Fig. 4d). However, when the time period of UV radiation was further extended, a further increase in ${{{\rm{I}}}_{{{{\rm{SO}}}}_{2}}}$ was not observed (Fig. 4e). It was evident that ${{{\rm{I}}}_{{{{\rm{SO}}}}_{2}}}$ exhibited a multilevel incremental trend similar to that of I_UV_ (Fig. 4f and Supplementary Table 5). The detailed comparison results showed that the nanochannels irradiated for ≥10 min yielded the most optimized detection effect, with an ${{{\rm{I}}}_{{{{\rm{SO}}}}_{2}}}$ value of up to 200 μA. The multistage increment feature confirmed that UV-driven increases in photoisomerization were responsible for efficiently boosting the generation of MC and overall SO_2_ detection performance. Importantly, unlike that in the liquid system, the effect of temperature on the reversible recovery of MC components on the membrane was minor (Supplementary Fig. 19). The good stability expanded the range of use of this nanodevice.

### Stability and preservation of SP/AAO nanosensor

**Fig. 5:**
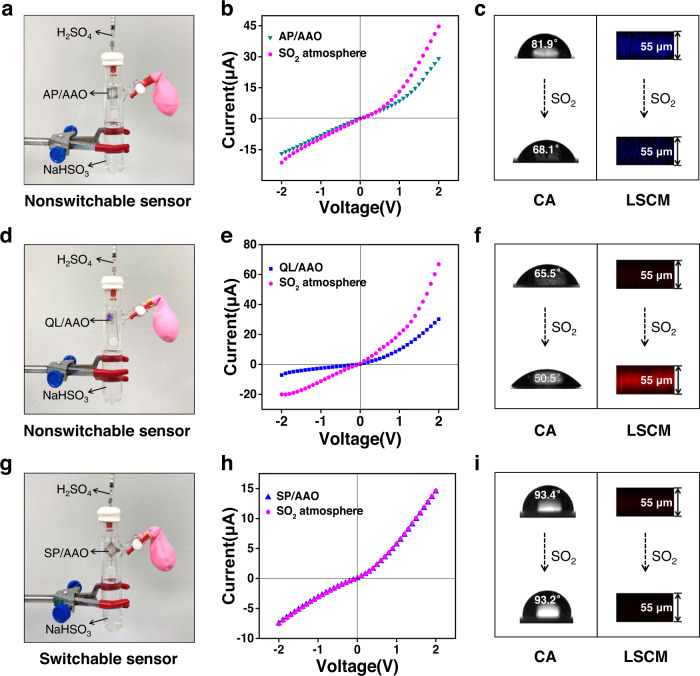
Stability and preservation of SP/AAO nanochannels. Construction of microconfined SO_2_ atmosphere for (**a**) AP/AAO nanochannels, (**d**) QL/AAO nanochannels, and (**g**) SP/AAO nanochannels. I–V curves of (**b**) AP/AAO nanochannels, (**e**) QL/AAO nanochannels, and (**h**) SP/AAO nanochannels before and after 4 days of exposure to the SO_2_ atmosphere. c, f, and **i** Corresponding CA change on the nanochannel surface and fluorescence change in the cross-sectional confocal images. AP/AAO aminobenzophenone/anodic aluminium oxide, QL/AAO quinolinium/anodic aluminium oxide, SP/AAO spiropyran/anodic aluminium oxide, CA contact angle, LSCM laser scanning confocal microscopy.

Stable product preservation, which facilitates large-scale production and provides data support for confirming packaging, storage conditions, and expiration periods, is another essential characteristic of a high-performance sensing device. To this end, an additional nonswitchable alternative device, an aminobenzophenone (AP)/AAO nanochannel platform (Supplementary Fig. 20), was fabricated and compared to our SP/AAO nanochannels. As a prerequisite, the reactivity of the nonswitchable nanochannels towards the SO_2_ solution was examined (Supplementary Fig. 21). The test results demonstrated the responsiveness of the nanochannels to the SO_2_ solution and the correctness of the nanodevice^34^. Then, to verify its stability in ambient air, a closed system for the production of SO_2_ gas was constructed using a two-way glass reaction tube, with the aim of shortening the experimental period. As shown in Fig. 5a, the container was filled with 1 mM NaHSO_3_ solution to provide trace SO_2_ gas. A syringe was used to inject dilute H_2_SO_4_ (2.5 mM) to accelerate the volatilization of SO_2_. The AP/AAO nanochannel sample was hung at the opening of the tube without contacting the aqueous solution. In this way, an artificial SO_2_-containing environment was simulated. It was found that the resulting nanochannels showed a higher ionic current and lower CA value after 4 days of exposure to the confined atmosphere (Fig. 5b, c). Furthermore, the blue fluorescence of the AP/AAO nanochannels was diminished upon reaction with the SO_2_ atmosphere (Fig. 5c), which may have been caused by the disruption of the original conjugation system of the aminobenzophenone probe (Supplementary Fig. 22). These phenomena suggested that the nonswitchable AP/AAO nanochannels reacted with the ambient SO_2_. To further study the stability of the nonswitchable device, another alternative quinolinium (QL)/AAO (Supplementary Figs. 23–26) nanosensor with a SO_2_-sensitive response was constructed^61^. This device was more easily destroyed by the SO_2_ atmosphere. When it was exposed to an atmosphere with a low level of SO_2_ (100 μM NaHSO_3_ and 250 μM H_2_SO_4_), we could still observe a clear increase in the ionic current and a decrease in the CA value after 4 days of exposure to the confined atmosphere (Fig. 5d–f). Moreover, an intense red fluorescence was observed in the cross-sectional LSCM images (Fig. 5f) due to an intramolecular charge transfer (ICT) effect in the product after exposure to SO_2_ (Supplementary Fig. 27)^61^. It was obvious that the surface chemical properties of these nonswitchable nanochannels have changed. In contrast, no changes in our SP/AAO system were observed due to the inertness of the structure and reactivity (Fig. 5g–i). Even after five months of placement, the SP/AAO nanochannels still exhibited the desired responsive behaviour and performance (Supplementary Fig. 28). Such comparative results suggested that the switchable SP/AAO device had a long storage life, while the nonswitchable nanochannels did not. Therefore, the proposed inert/active switchable SP/AAO nanosensors demonstrated their potential for long-term preservation and on-demand detection.

### Selectivity, response rate, and linear quantification

**Fig. 6:**
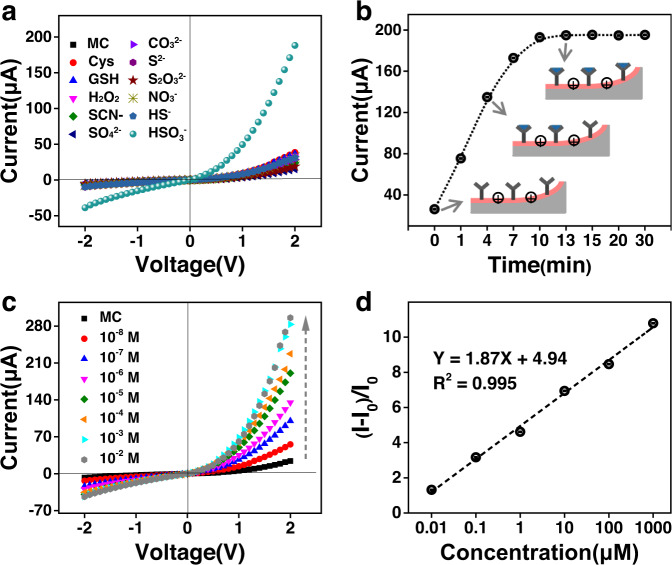
Performance of SP/AAO nanochannels in SO_2_ detection. **a** Current changes for different analyte treatments. **b** Time-dependent current changes. **c** Concentration-dependent current changes. **d** Relationship between ionic current ratios (I_0_ − I)/I_0_ and SO_2_ concentrations. MC merocyanine, Cys cysteine, GSH glutathione.

Having demonstrated proof-of-concept of the on-demand response function, the selectivity and specificity of the UV-activated SP/AAO sensor towards SO_2_ were further evaluated. For this purpose, some representative competitive species (such as thiols, H_2_O_2_, and anions) were chosen to treat the UV-activated SP/AAO nanochannels. As shown in Fig. 6a, the SP/AAO nanosensor showed a minor current response towards the other interfering analytes. In contrast, an enormous increase in the 2 V current was observed only in the presence of SO_2_. This discrepancy indicated that the nanochannel sensor had much higher selectivity for SO_2_ over other analytes at the same 10 μM concentration. This could be ascribed to the strong nucleophilic ability of SO_2_, which could attack the electron-rich C ═ C in MC more efficiently to generate new stable adducts.

The time-dependent I−V changes of the SP/AAO nanochannels were also monitored. When in contact with 10 μM SO_2_, the ionic current at 2 V increased considerably along with the response time (Supplementary Fig. 29). As a result, the time-dependent ionic conductance properties could be observed distinctly from the current–time traces at a constant voltage of 2 V (Fig. 6b). It was clear that a rapid increase in ionic current at 2 V was observed during the first 10 min. Then, at ≥13 min, the ion current levelled off at ~195 μA, indicative of the saturation of the nucleophilic addition reaction. The overall trend of the current–time traces demonstrated that the reaction in the nanosystem reached equilibrium at ~13 min. During this SO_2_ reaction, MC essentially did not switch back to SP in a darkroom or a bright laboratory environment (Supplementary Fig. 30), indicating the feasibility of the SO_2_ response of the switchable nanochannels. Statistical data of these current signals at different times were finally provided to demonstrate the repetitive nature of the time-dependent response (Supplementary Table 6).

Afterwards, the concentration-dependent SO_2_ response of the SP/AAO nanochannels was characterized by I–V measurement (Fig. 6c). The ionic current at 2 V increased considerably with increasing SO_2_ concentration, but the current increase at −2 V was low. As a result, the rectification ratio was progressively enhanced with great repeatability (Supplementary Fig. 31 and Supplementary Table 7). Such a phenomenon was attributed to the continuous production of MC−SO_3_H adducts leading to increased hydrophilic/hydrophobic asymmetry. Importantly, our SP/AAO nanochannels with a nanoconfinement barrier layer rendered the SO_2_ sensor sensitive detection capabilities by the amplified rectifier currents. As shown in Fig. 6c, even at SO_2_ concentrations as low as 10 nM, the nanochannel sensor responded sensitively, yielding a high ${{{\rm{I}}}_{{{{\rm{SO}}}}_{2}}}$ of ≈31.6 μA at 2 V. When exposed to 1 mM SO_2_, the system’s current was enhanced to ~283 μA at 2 V, resulting in a high rectification ratio of approximately 6.52. No further increase in current was detected upon exposure to a higher concentration of SO_2_. This confirmed that all C ═ C nucleophilic sites were completely saturated with SO_2_. Unlike the rectifying response behaviour of tubular nanochannels, the cylindrical nanochannels showed linearly symmetric I−V curves and no significant response current (Supplementary Fig. 32) due to the absence of the asymmetric structure and confinement effect. In addition, with our SP/AAO detection system, there was a good linear relationship (R^2^ = 0.995) between the current change ratio (I_0_ − I)/I_0_ and SO_2_ concentration over a wide range of 10 nM–1 mM, which was better than that of reported probes for the detection of SO_2_ (Supplementary Table 8). I_0_ and I represent the ionic current value at 2 V before and after reacting with SO_2_ (Fig. 6d). Additionally, we used the platform to determine the SO_2_ content in the red wine and shiitake mushroom. As shown in Supplementary Fig. 33, the 2 V ion current of the nanochannels was significantly enhanced after treatment with red wine or a solution extracted from shiitake mushroom. Based on the above linear relationship, the SO_2_ concentrations in the red wine and shiitake mushroom were calculated to be ~1 mM and 56 μM, respectively. Thus, the designed nanochannel device has great potential for the quantitative detection of SO_2_.

## Discussion

In summary, we developed a finely tuneable light-controlled nanochannel sensor with an inert/active state-switchable photochromic property for on-demand recognition of SO_2_. Here, asymmetric tubular AAO nanochannels containing nanoporous arrays and sealed barrier layers were used as rigid backbones to maximize nanoconfinement effects to realize rectification properties. In this structure, photochromic SP probes were delicately anchored on the porous side of the nanochannels for enhanced response performance. Through the conversion of the SP probes to their MC photoisomers upon UV irradiation, the inert nanochannel system was activated for SO_2_ detection to promote long-term storage and unbiased real-time response. As a result, an on-demand nanochannel sensor with SO_2_ response was established, and the expected light-regulated response ability was realized. This switchable feature significantly improved the device’s stability compared to the nonswitchable feature, which is beneficial for long-term storage. The newly developed nanosensor exhibited high selectivity, excellent sensitivity, and a wide linear detection range from 10 nM to 1 mM. We believe that this SP/AAO nanochannel system could constitute a proof-of-concept for a light-operated inert/active state-switchable sensor and could be used in a wide range of practical applications, such as reactivity switchers, locked nanosensors, and on-demand detectors. This work provides a reference for developing switchable nanosensors under other stimuli and facilitates their application in the field of target detection.

## Methods

### General

All reagents were commercially available and purchased from Sigma‒Aldrich. They were used as supplies without further purification. Tubular and cylindrical anodic aluminium oxide (AAO) nanochannels were purchased from Puyuan Nanotechnology Co., Ltd. UV light was supplied by a camera obscura three-purpose ultraviolet analyser (WFH-203B, Shanghai Jingke Industrial Co., Ltd). The radiation wavelength was 365 nm, and the radiation power was approximately 1.5 mW. The irradiation power was measured using a Coherent FieldMate Laser Power Metre 1098297. The results of the bar graphs were expressed as the means ± SDs. Statistical analysis was performed with GraphPad Prism 8. Experiments were repeated at least three times with the representative data shown.

### Synthesis and characterization

Synthesis and relevant characterization details are provided in the Supplementary Information.

### Immobilization of SP

The SP probe was covalently immobilized on the interior surface of the AAO nanochannels via a coupling reaction (Supplementary Fig. 1). First, the AAO nanochannels were immersed in an ethanol solution of (3-aminopropyl)triethoxysilane (APTES, 1:4 by vol) for 10 min and then thoroughly washed with ethanol. The resulting membrane was placed in an oven for 1 h to better crosslink the silane and alumina. Additionally, an ethanol solution of SP (10 mM), 1-ethyl-3-(3-dimethylaminopropyl) carbodiimide hydrochloride (EDC, 10 mM), and N-hydroxysuccinimide (NHS, 10 mM) was reacted for 1 h to obtain a carboxyl-activated SP probe solution. It showed a clear pale pink colour. Next, the porous side of the APTES-modified alumina nanochannel membrane was exposed to the probe solution above, while the other side (barrier layer) was exposed to pure ethanol solution. The modification method was defined as diffusion-limited patterning (DLP) conducted with a special H-shaped tank at room temperature and pressure (Supplementary Fig. 6). After specified modification times of 1, 2, or 3 h, the membrane was rinsed many times with ethanol to remove the free probe molecules. Finally, all of the resulting membranes were washed with Milli-Q water (18.2 MΩ, Milli-Q Academic A10, USA) before the current–voltage (I–V) measurements.

### Ion current measurements

The ion conduction and rectification characteristics of the AAO nanochannels, APTES-treated nanochannels, and SP/AAO nanochannels under UV/Vis irradiation were characterized by measuring I−V curves. The obtained nanochannel membrane, as a middle separator, was mounted between two chambers of the electrochemical tank, each of which was filled with 0.1 M KCl electrolyte at pH 5.5 and one Ag/AgCl electrode was installed vertically (Supplementary Fig. 34). Transmembrane I−V curves were recorded using a Keithley 6487 picoammeter and a control computer. The picoammeter served as a voltage source to drive transmembrane ionic transportation. The scanning voltage was varied from −2 to +2 V with 100 mV steps. Herein, the bottom side of the AAO was determined to have a negative potential, while its opposite opened pore side was defined to have a positive potential. Measurement of the UV response was conducted after various time periods of UV irradiation (365 nm, 1.5 mW). Measurement of the SO_2_ response was performed after UV-activated SP/AAO nanochannels were immersed in specific concentrations of sulfur dioxide (SO_2_). Sodium hydrogen sulfite (NaHSO_3_) was used as the SO_2_ donor and generator.

## Supplementary information

Supplementary Information

## Data Availability

The data that supports the findings of this study can be found in the manuscript, its Supplementary Information, or are available from the corresponding author upon request.
