# Supplementary material for: Switchable biomimetic nanochannels for on-demand SO2 detection by light-controlled photochromism
Source: Nat Commun. 2023 Apr 5;14:1901. doi: 10.1038/s41467-023-37654-y (PMC10076267; doi:10.1038/s41467-023-37654-y)
Supplement: Supplementary file 1 — Supplementary Information [file 41467_2023_37654_MOESM1_ESM.pdf]

Supplementary Information

Switchable biomimetic nanochannels for on-demand SO_2 detection by light-controlled photochromism

Dan Zhang, Yongjie Sun, Zhichao Wang, Fang Liu & Xuanjun Zhang*

Supplementary Methods

Synthesis of SP. The spiropyran (SP) probe was facilely synthesized by a slight modification to the preparation procedure provided in the previous article (Supplementary Fig. 2).[S1] Step 1: 2,3,3-trimethyl-3H-indole (3.18 g, 20 mmol) and 3-iodopropionic acid (4.00 g, 20 mmol) were dissolved in 30 mL of acetonitrile and the mixture was reacted at reflux overnight at 75 °C. After completion of the reaction, the solvent was spun dry under reduced pressure. The residue was washed three times with anhydrous ether and then dissolved with acetone to obtain a purplish-red suspension. Finally, the suspension was filtered and washed with acetone to obtain a pink powder with a yield of 68.9%. It was defined as an intermediate product. Step 2: The intermediate product (1.80 g, 5 mmol), 5-nitrosalicylaldehyde (0.92 g, 5.5 mmol), and triethylamine (0.83 mL, 6 mmol) were dissolved in 40 mL of anhydrous ethanol, and the mixture was reacted with heating reflux overnight. The reaction process was protected from light. After the reaction was completed, the reaction solution was cooled to room temperature. The precipitate was filtered, washed with ice ethanol, and dried to obtain an earthy yellow powder with an 83.7% yield. It was defined as an end product SP.

Synthesis of QL. The quinolinium (QL) probe was facilely synthesized by a slight modification to the preparation procedure provided in the previous article (Supplementary Fig. 23).[S2] Step 1: 3-Acetylquinoline (3.42 g, 20 mmol) and 3-iodopropionic acid (4.00 g, 20 mmol) were dissolved in 30 mL of acetonitrile and the mixture was reacted at reflux overnight at 85 °C. After completion of the reaction, the solvent was spun dry under reduced pressure. The residue was washed three times with anhydrous ether and then dissolved with acetone to obtain a yellow suspension. Step 2: The intermediate product (483 mg, 1.3 mmol), 2-(4-(Diethylamino)-2-hydroxybenzoyl) benzoic acid (407 mg, 1.3 mmol), and concentrated H_2SO_4 (98%, 7 mL) were reacted at 90°C for 4 h. After the reaction was completed, the solution was first cooled to room temperature and poured into ice water (50 mL). Then $HClO_4$ (70%, 1.5 mL) was added dropwise to the mixed solution. The obtained precipitate was filtered and washed several times with ice water. Finally, the crude product was purified by a silica gel column (CH_2Cl_2/CH_3OH = 20/1) to obtain a deep purple powder with a 57.1% yield.

Characterizations. Probe structure and probe response were determined by using hydrogen nuclear magnetic resonance spectrum (^{1}H NMR) and mass spectrometer (MS). The morphology of the tubular anodic aluminium oxide (AAO) nanochannels was characterized using a field emission scanning electron microscope (Zeiss Sigma FESEM, Germany). Water contact angles (CA) were measured using a SCI4000 automatic contact angle measuring instrument (China). Element component analyses and Zeta potentials were analysed by a PHI 5000 VersaProbe III XPS system equipped with a monochromatic Al Kα X-ray source (Kanagawa, Japan) and a SurPASS instrument (Anton Paar GmbH, Austria), respectively. Confocal images from cross-sectional views were performed on Carl Zeiss LSM710 confocal microscope (Carl Zeiss Pty Ltd, Germany). The excitation wavelength of the laser was set to 543 nm.

Supplementary Discussion

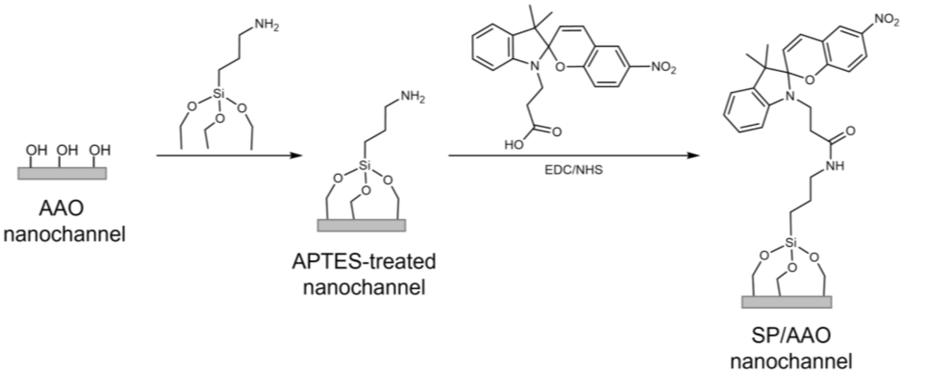

Supplementary Fig. 1 The modification of SP probes on the inner surface of AAO nanochannels.

The tubular AAO nanochannels with abundant hydroxyl groups reacted with APTES by a covalently grafted procedure, resulting in a silanized AAO surface with terminal amino groups. Then, SP molecules with carboxyl groups were grafted to an APTES surface by classical coupling reaction with help of 1-ethyl-3-(3-dimethylaminopropyl) carbodiimide hydrochloride (EDC) and N-hydroxysuccinimide (NHS). Therefore, the SP functionalized nanochannels were finally obtained and its end showed a neutral spirocyclic group. Under UV irradiation, the C−O pyran bond opened and at pH 5.5 photo-isomerized to the charge-separated zwitterionic MC structure incorporating a C=C fragment. Such electron-enriched double bonds offered a nucleophilic coordination site for various nucleophile analytes, such as SO_2 and biological thioalcohol.

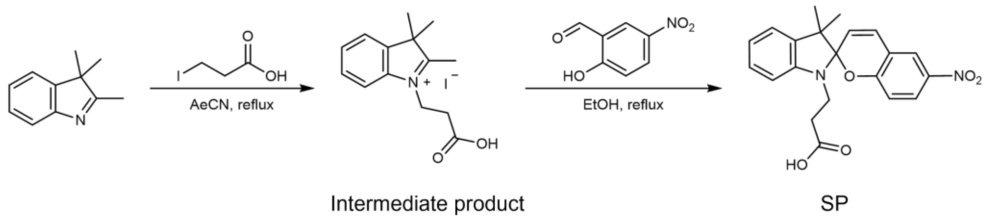

Supplementary Fig. 2 The synthesis route of carboxy-terminal SP probe.

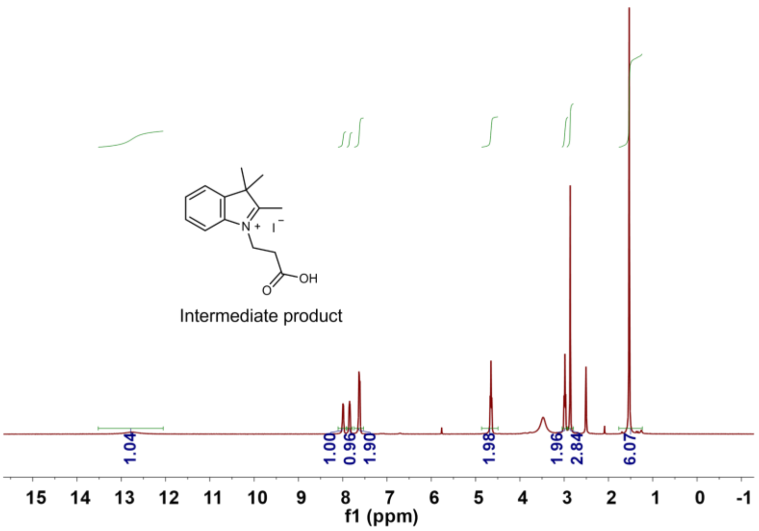

Supplementary Fig. 3 ^{1}H NMR of intermediate product.

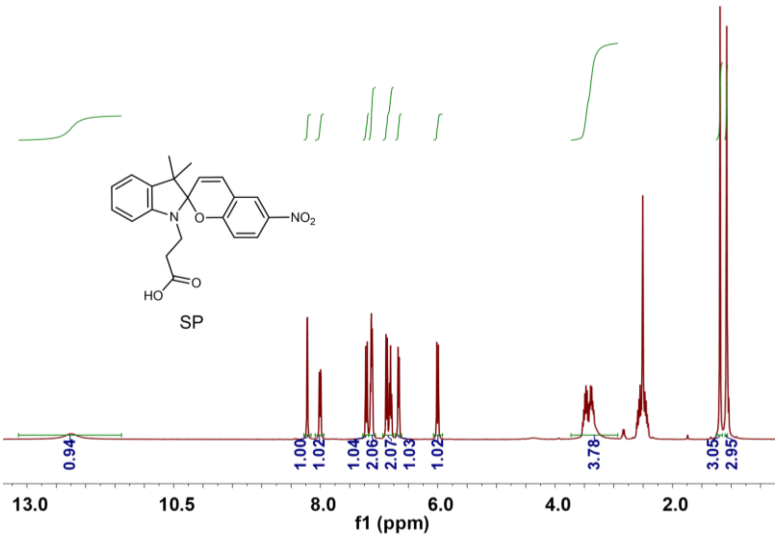

Supplementary Fig. 4 ^{1}H NMR of SP probe.

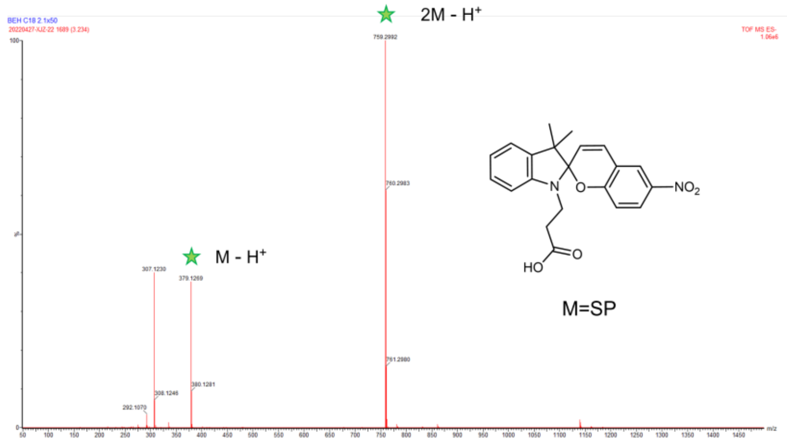

Supplementary Fig. 5 MS of SP probe.

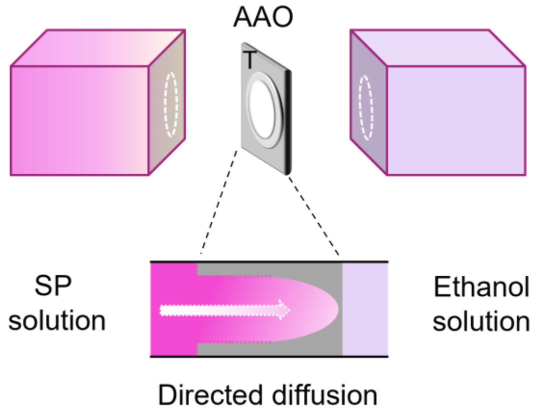

Supplementary Fig. 6 The modification of SP probes to the aminated surface of AAO nanochannels using the diffusion-limited patterning method (DLP). The ethanol solution of SP probes was arranged on the porous side, while pure ethanol solution was arranged on the opposite barrier layer side.

The diffusion-limited patterning (DLP) model is a unique technique for patterning functionalized molecules on surfaces of the nanofluidic channels.[S3] The modification was realized by the diffusion of the functional molecules in a solution phase. Herein, an APTES-treated AAO membrane with tubular nanochannel geometry was mounted between the two chambers of the H-shaped electrochemical tank (Supplementary Fig. 6). The SP ethanol solution (containing EDC and NHS) was added to the porous side of AAO, while pure ethanol solution was added to the opposite barrier layer side. In this case, a concentration gradient was built along the nanochannel axis from the porous side to the barrier layer. As a result, SP molecules could spread from a high concentration (porous side) to a low concentration (barrier layer side). During the process of diffusion, the SP molecules could be chemically conjugated with the inner surfaces of the nanochannels, leading to the successful patterning of functionalized SP to the surface of AAO nanochannels. Herein, SP molecules were successfully immobilized onto a porous segment of the tube-shaped AAO nanochannels, but not on the blocking layer side. This results in the formation of asymmetric SP/AAO nanochannels. Importantly, by adjusting the time of patterning, SP/AAO nanochannels' surface properties including surface charge and surface wettability could be exactly controlled.

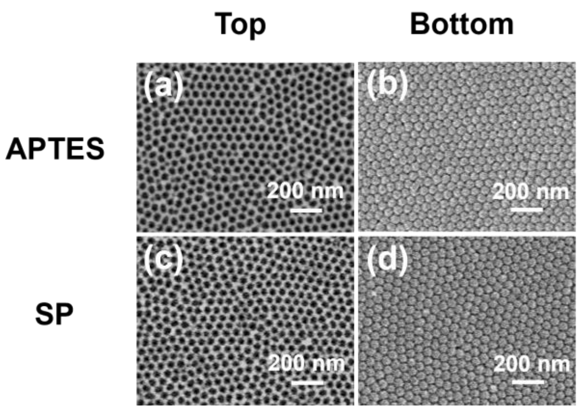

Supplementary Fig. 7 SEM images of the top and bottom surfaces of the AAO nanochannels modified by (a, b) APTES and (c, d) SP molecules. In the APTES-modified AAO and SP/AAO nanochannels, we could observe a porous structure on the top surface and a dense hemispherical dome structure on the bottom surface, which was similar to the pristine AAO. It was concluded that the AAO nanochannels were not damaged during the attachment process.

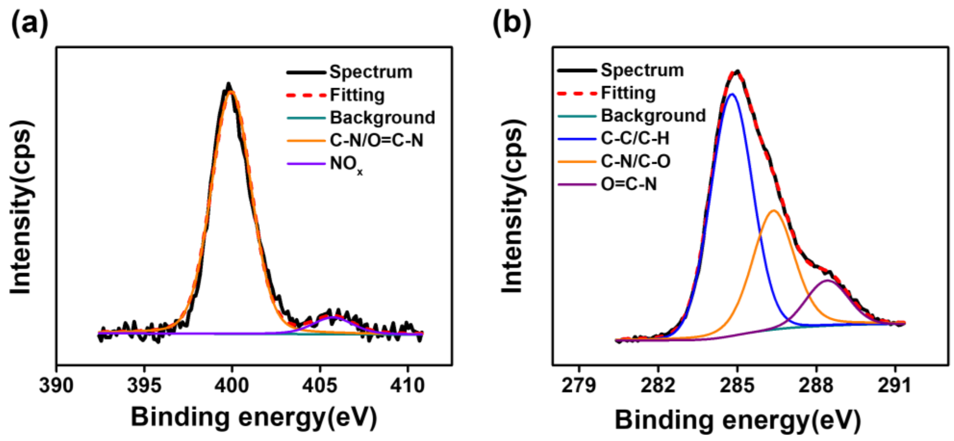

Supplementary Fig. 8 Detailed deconvoluted X-ray photoelectron spectra of (a) N1s and (b) C1s for the SP/AAO nanochannels.

X-ray photoelectron spectra (XPS) is a versatile surface analysis technique for determining the elemental composition information of the materials. Here, detailed deconvolution XPS spectra of N1s and C1s were applied to identify the successful chemical immobilization of SP on the APTES-treated AAO nanochannels.[S4] The curve fitting was performed by PHI MultiPak software, and Gaussian-Lorentz functions and Shirley background were used. As shown in Supplementary Fig. 8a, the deconvoluted N1s spectrum showed two characteristic peaks at 399.9 eV and 405.8 eV, which are assigned to C–N/O=C–N bonds from the indoline/amide nitrogen and N–O bonds from the nitro group, respectively, indicative of the presence of SP components as expected. However, no characteristic peak of the ring-opening MC indoline fraction (N^+ component) was observed at 400.5-401.0 eV.[S5,S6] These results suggested that the SP probes on the SP/AAO nanochannels had not translated into MC form during XPS measurements.

In the C1s core level region of Supplementary Fig. 8b, the XPS C1s spectra were fitted into three peaks at 284.8 eV, 286.4 eV, and 288.4 eV, which are associated with C–C/C–H, C–N/C–O, and O=C–N groups, respectively. The high-resolution C1s peak from SP/AAO nanochannels confirmed the chemical grafting of SP probes, consistent with the above N1s result.

It is worth noting both the nitrogen peak found at approximately 399.9 eV and the carbon peak found at approximately 288.4 eV for the SP/AAO membrane belonged to the amide groups.[S7-S9] This indicated the covalent functionalization of SP probes on APTES-modified AAO surface via EDC-NHS chemistry. All of these results verified that SP probes were successfully immobilized onto the channels by covalent binding.

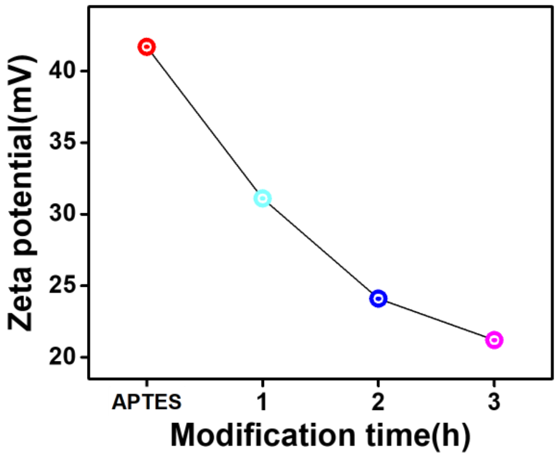

Supplementary Fig. 9 Zeta-potential changes of the AAO nanochannels at different modification times of SP probes.

Zeta potential is a model parameter describing the surface charged behaviour including electrical properties and the number of charges at the solid-liquid interface. As the data in Supplementary Fig. 9 demonstrated, the APTES treated-AAO nanochannels exhibited the highest positive values in zeta potential. The results indicated that abundant positive charges on the nanochannel surface appeared, which should be attributed to the protonation of the terminal amino groups from APTES in the slightly acid environment. Then the zeta potential of the SP/AAO nanochannels decreased with the increase of SP modification time from 0 to 3 h. This trend indicated that neutral SP components were gradually deposited on APTES-treated AAO as the modification time extended. As a result, the amino positive charge of APTES and the hydroxyl positive charge of AAO would be gradually covered, resulting in a decreasing positive charge density. Benefiting from the confinement effect in nanoscale channels, the reduction of the positive charge density would degrade the ion selectivity and ion rectification of the nanosystem. The overall zeta potential trend well verified the surface charge variation and ion transport behaviour across the nanochannel membrane.

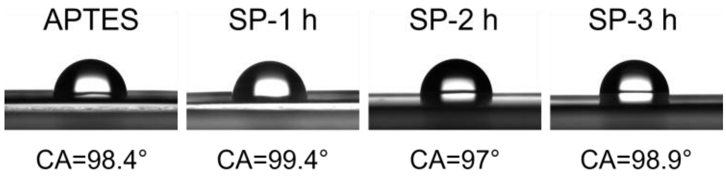

Supplementary Fig. 10 CA of the bottom surface of APTES-treated AAO nanochannels at different modification times of SP probes. The CA of the barrier layer on the bottom surface is less changed in the process of patterning SP to the nanochannel surface using the DLP method. This is because the SP solution on the porous side cannot diffuse to the blocking layer side of AAO due to the closed morphology of the blocking layer.

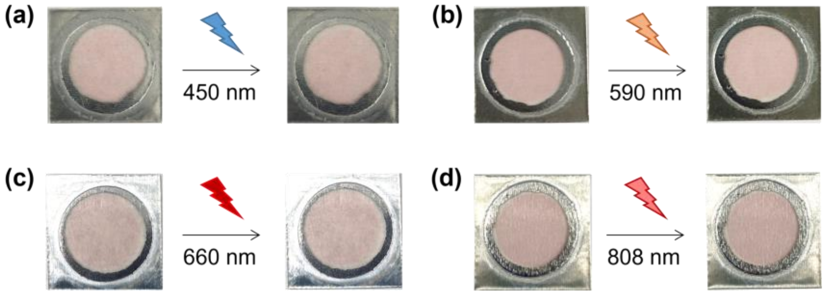

Supplementary Fig. 11 Optical photograph of the SP/AAO nanochannels after 10 minutes of exposure to (a) blue (450 nm, 20 mW), (b) yellow (590 nm, 6.4 mW), (c) red (660 nm, 12.6 mW), and (d) infrared light (808 nm, 14.5 mW). After 10 minutes of visible and infrared light irradiation, no colour change was observed in the SP/AAO nanochannels, suggesting that UV irradiation did not convert SP molecules to the MC form.

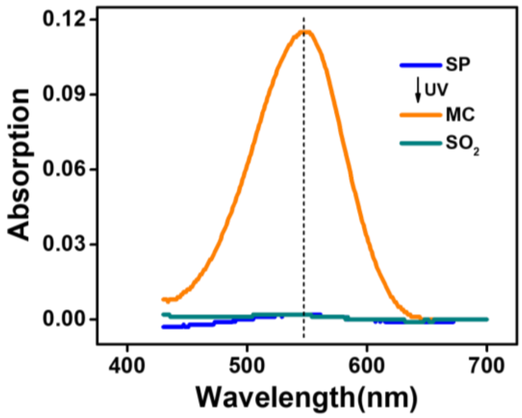

Supplementary Fig. 12 The absorption changes of SP/AAO nanochannels during UV irradiation and SO_2 exposure.

Before irradiation, the spiropyrane molecules were predominantly in the closed-ring SP form. As shown in Supplementary Fig. 12, the SP-state nanochannels did not have any absorption band in the visible region. After the UV stimulated conversion to the MC purple isomer, the MC-state system incorporated a charge-separated zwitterionic conjugated structure. At this point, we observed a high absorption peak at 550 nm, which is a characteristic peak of the MC.[S10] Subsequently, when in contact with SO_2, the peak at 550 nm fully disappeared. This was because the formation of the new MC−SO_3H species destroyed the conjugated structure and electron configuration of MC. Such absorption phenomena are consistent with changes in contact angle, fluorescence, and optical colour during UV irradiation and SO_2 exposure. Together, the results demonstrated that UV stimulus could control the photoconversion of SP to MC enabling SO_2 binding into the nanochannel. Therefore, the as-prepared SP/AAO nanochannels could be exploited for real-time signalling of SO_2 for on-demand sensing applications in a light-controlled manner.

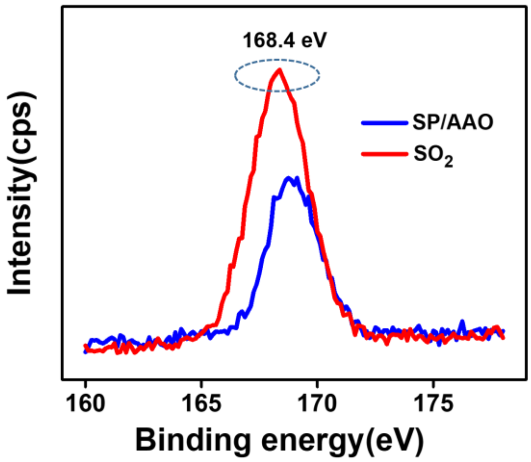

Supplementary Fig. 13 S2p peak of X-ray photoelectron spectra for the UV-activated SP/AAO nanochannels before and after interaction with SO_2.

As demonstrated in Supplementary Fig. 13, before and after the SO_2 response, we observed a peak on the SP/AAO nanochannels with a binding energy of about 168.4 eV, which is the characteristic peak for S2p elements.[S11] It is important to explain that the sulfur element that appears before the SO_2 addition originated from the sulfuric acid electrolyte used in the preparation of the AAO nanochannels. However, after being treated with SO_2, a significant increase in the sulfur peak was observed. As presented by XPS atomic ratio, the S atomic percentage in the SP/AAO nanochannel increased from 1.02 to 1.83 after the reaction with SO_2 (Supplementary Table 2). The result is further proof of the successful nucleophilic attack of SO_2 on MC components of the functionalized nanochannels.

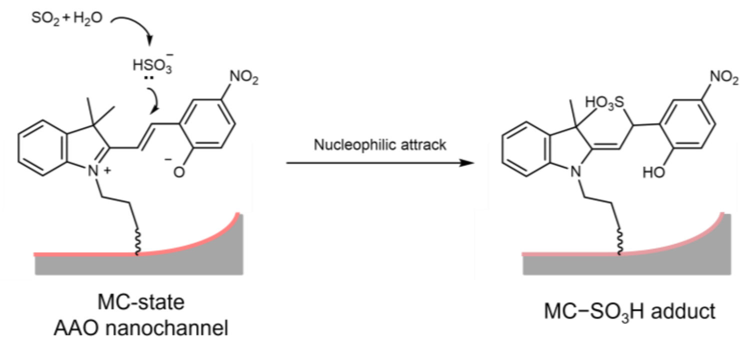

Supplementary Fig. 14 Reaction mechanism between SO_2 and C=C of the MC form in the nanochannels.

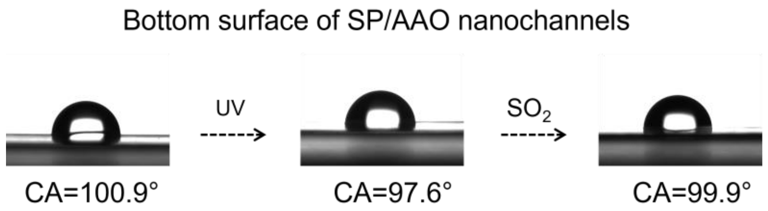

Supplementary Fig. 15 CA of the bottom surface of SP/AAO nanochannels during UV-controlled response to SO_2. After UV stimulus and SO_2 detection, no changes in CA at the bottom blocking layer were observed due to the lack of SP components and response behaviour on the bottom surface.

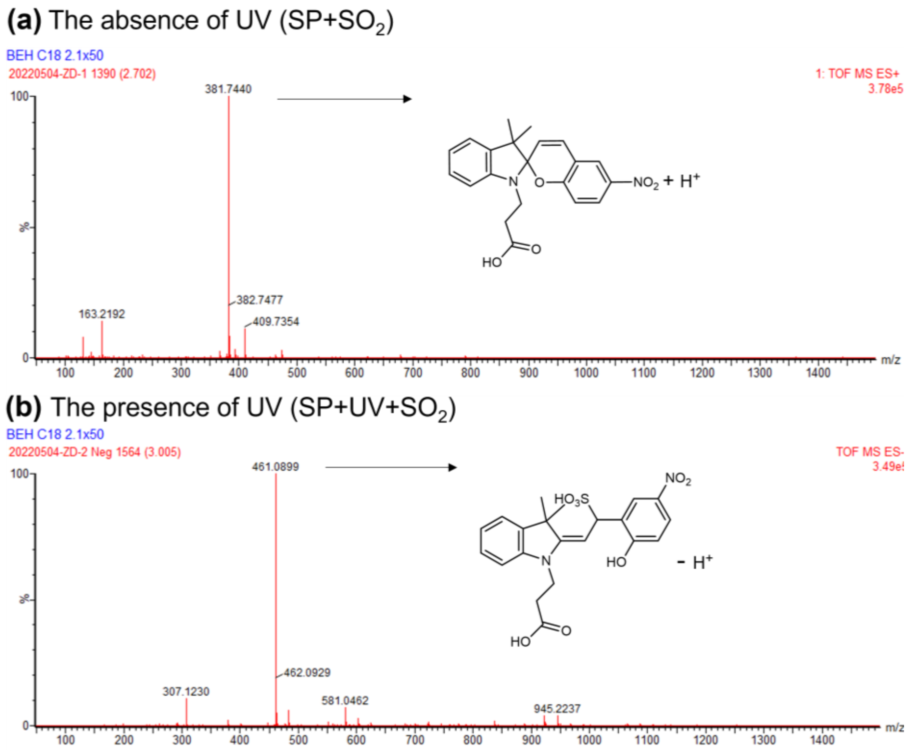

Supplementary Fig. 16 The MS analysis of the reaction between SP and SO_2 in solution (a) in the absence of UV and (b) in the presence of UV.

To further demonstrate that the light-controlled inert/active switching function can be used for on-demand detection of SO_2, the response of the SP probe and SO_2 with and without UV irradiation was characterized by using mass spectrometry in solution. As shown in Supplementary Fig. 16a, the resulting mass spectrum in the absence of UV irradiation exhibited a peak at m/z = 381.7440, which was attributed to the SP. The results clearly showed that no new product was formed between SP and SO_2, i.e., the original SP was chemically inert to SO_2. However, when the SP solution was subjected to UV irradiation prior to the SO_2 treatment, we observed a new peak at m/z = 461.0899 indicative of [MC−SO_3H - H^+] (Supplementary Fig. 16b). The generation of the MC−SO_3H compound after UV illumination confirmed the addition reaction between SO_2 and the photoisomer MC of SP. Accordingly, the comparative results of (a) and (b) provided evidence for the successful light-controlled reaction behaviour of SP towards SO_2. It was concluded that the specific reactivity of MC to SO_2, but not SP to SO_2.

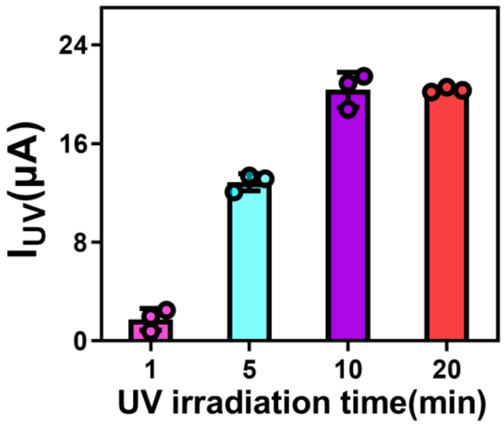

Supplementary Fig. 17 UV response current (I_{UV}) of SP/AAO nanochannels at different times. Error bars denote the standard deviation from three different samples.

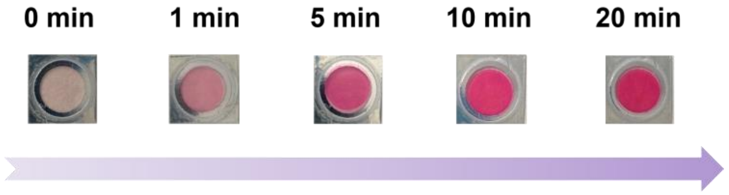

Supplementary Fig. 18 Optical photograph of SP/AAO nanochannel membrane at incremental UV irradiation times. As the UV radiation time increases from 0 to 10 min, the colour of the membrane gradually changed from colourless to pink to purple. However, the colour of the nanochannels stopped changing after 10 min. This intuitionistically reflected the photochromic shift of the SP/AAO nanochannels and reached stability within 10 min. The result was consistent with the trend of the UV response current.

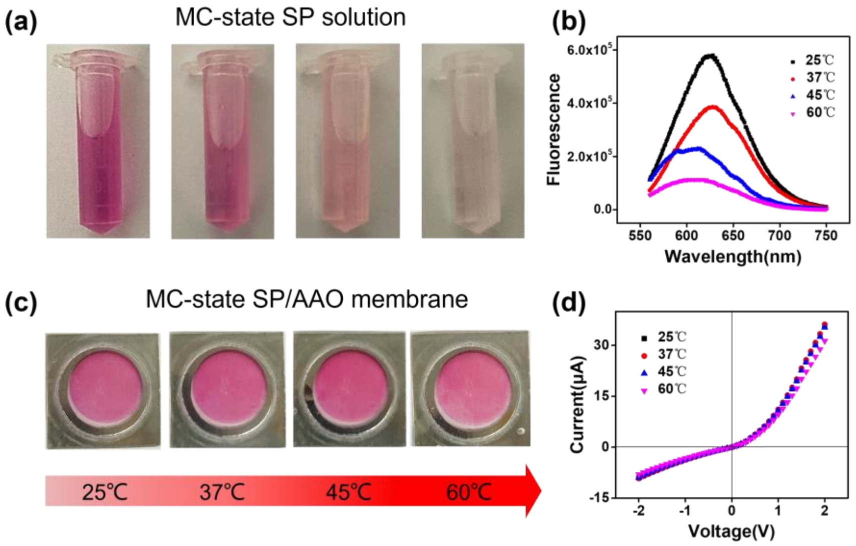

Supplementary Fig. 19 Effect of temperature on (a, b) MC-state SP solution and (c, d) MC-state SP/AAO nanochannel membrane.

It has been reported that heating and/or visible light irradiation can convert MC back to SP.[S12] Firstly, we tested the effect of temperature on liquid MC in the darkroom. The optical photographs clearly demonstrated the gradual fading of the colour of MC solution with increasing temperature from 25, 37, 45 to 60°C (Supplementary Fig. 19a). This phenomenon verified that the coloured MC reverted to colourless SP under heating conditions. In addition, the fluorescence data further proved this effect. As shown in Supplementary Fig. 19b, the fluorescence intensity decreased as the temperature increased, indicating that heating led to a gradual reduction of the MC component in the solution.

However, once the molecules were immobilized on the AAO substrate, the MC components were relatively stable under heating conditions. As in Supplementary Fig. 19c, there is no significant colour change on the surface of the MC-state SP/AAO nanochannels at different temperatures of 25, 37, and 45 °C. The approximate effect of heating on the ion transport behaviour implied that the surface composition of the channels is in a similar state in all three cases (Supplementary Fig. 19d). As the temperature increased to 60°C, we observed a slight discoloration of the membrane. At this point, a small difference in ionic current across the membrane was recorded. This should be due to the return of a tiny amount of MC on the nanochannels to the less

hydrophilic SP state. These phenomena indicated that temperature made little influence on the solid SP/AAO nanochannel sensor. The overall comparative results showed that our SP/AAO nanochannels can be applied in a wide range of SO_2 detection than liquid probes.

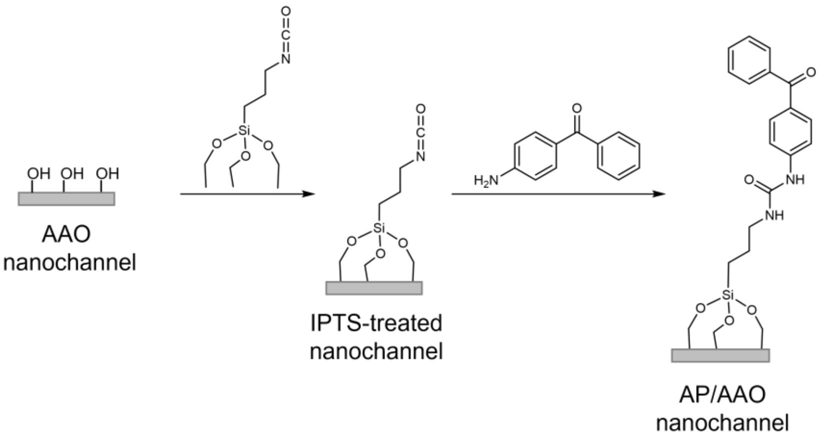

Supplementary Fig. 20 The modification of AP probes on the inner surface of AAO nanochannels to build a nonswitchable AP/AAO nanochannel sensor.

First, 3-isocyanopropyltriethoxysilane (IPTS, another silane coupling agent with bilateral functional groups) was modified to the inner surface of the AAO channel. Its terminal isocyanic acid was able to react with the amino group. Thus, the AP molecules with the amino groups could be chemically immobilized on the IPTS surface using the DLP technique. The diffusion time was set to 10 h to achieve uniform modification.

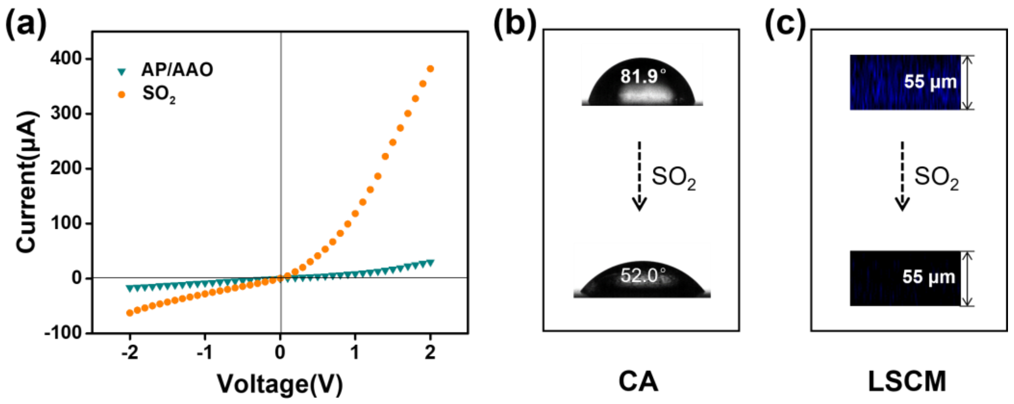

Supplementary Fig. 21 The response performance of the AP/AAO nanochannels to SO_2 solution.

In 2020, Sun et al. developed a high-efficiency bisulfite nanosensor by immobilizing aminobenzophenone (AP) on the wall of a single conical nanochannel in a polyethylene terephthalate (PET) membrane.[S13] After completion of immobilization, the appearance of blue fluorescence on the cross-section of the nanochannel proved the successful modification of the AP probes. The ketone group of the probes on the biosensor surface could specifically interact with HSO_3^- via a nucleophilic reaction, followed by a significant increase in the hydrophilicity and ion transportation.

Simulating the intelligent nanofluidic biosensors described above, we constructed here a porous AP/AAO nanochannel system. As shown in Supplementary Fig. 21, a blue fluorescence signal appears in the AP/AAO nanochannels, which was direct evidence of the successful modification of AP probes. The SO_2 response result also described a similar trend. After HSO_3^- solution treatment, we observed a significant increase in the ionic current and surface wettability of AP/AAO nanochannels (Supplementary Fig. 21a, b). In addition, the response behaviour of AP and SO_2 in the nanochannel spaces was tracked by LSCM. As shown in Supplementary Fig. 21c, the fluorescence signal decreased dramatically after the reaction. This indicated that SO_2 could efficiently attack the ketone group on the probe, leading to the disruption of the conjugated structure of the AP. In conclusion, these combined results demonstrated the successful construction of an AP/AAO nanochannel sensor for SO_2 detection.

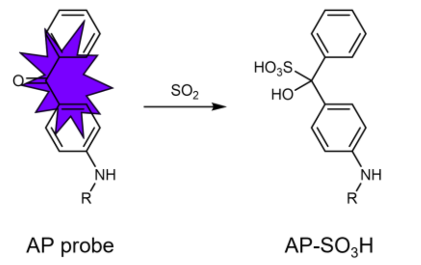

Supplementary Fig. 22 The disruption of the original conjugation system of the AP probe in response to SO_2.

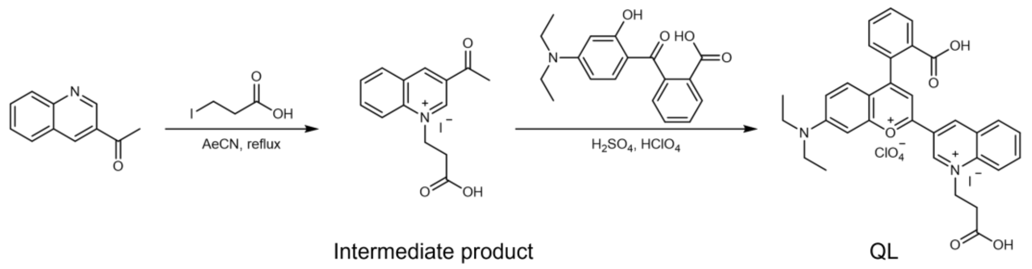

Supplementary Fig. 23 The synthesis route of carboxy-terminal QL probe.

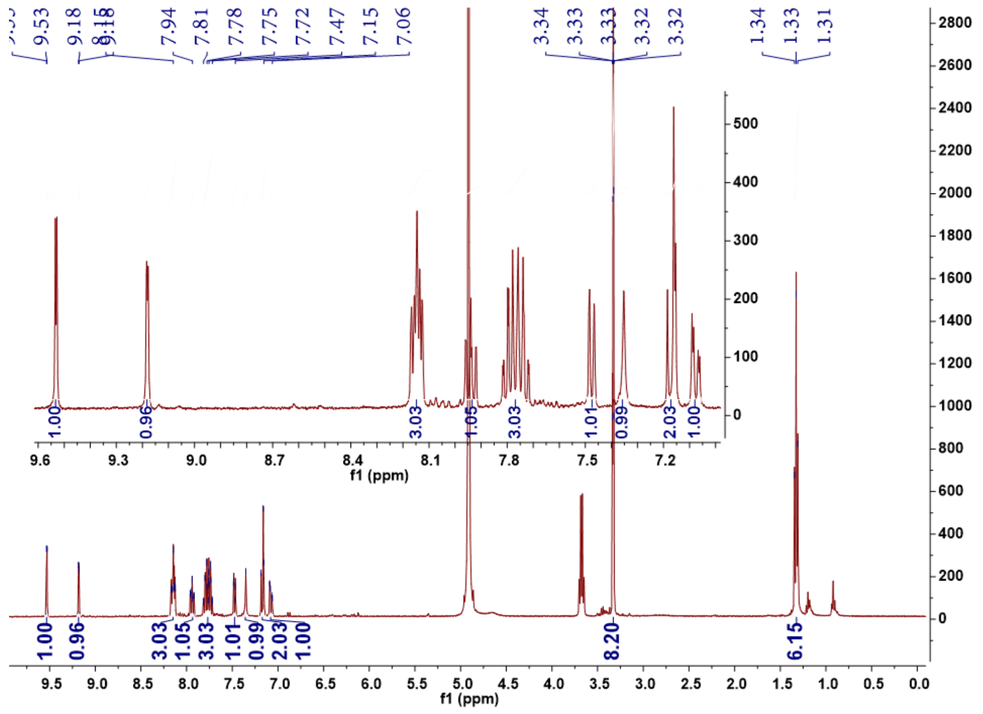

Supplementary Fig. 24 ^{1}H NMR of QL probe.

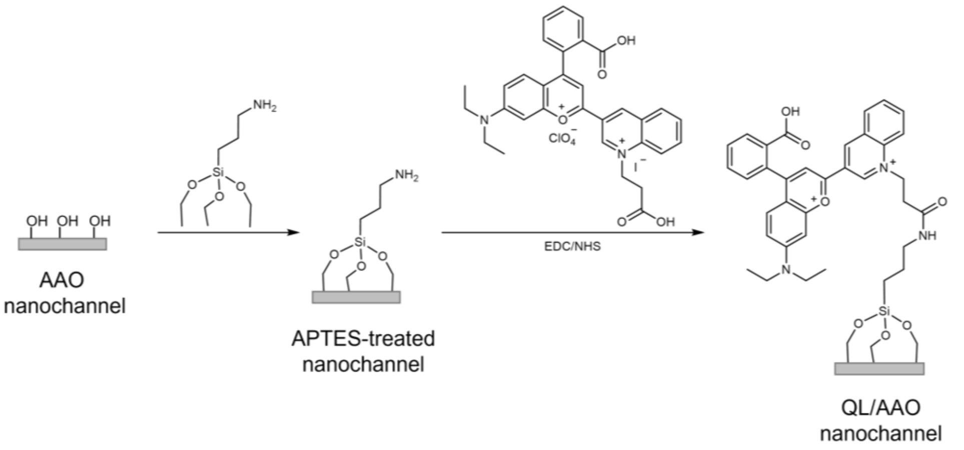

Supplementary Fig. 25 The modification of QL probes on the inner surface of AAO nanochannels to build a nonswitchable QL/AAO nanochannel sensor.

The modification for silanization is the same as this step for the construction of SP/AAO nanochannels. Similarly, QL molecules with carboxyl groups were grafted to an APTES surface by classical coupling reaction with help of EDC and NHS using DLP technology. The difference is that for QL probes, the diffusion time is set to 10 h.

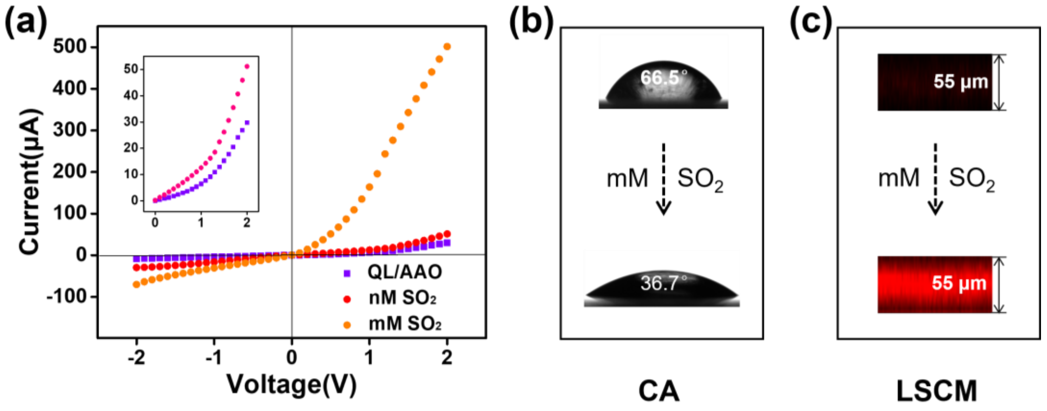

Supplementary Fig. 26 The response performance of the QL/AAO nanochannels to SO_2 solution.

After responding to 1 mM SO_2, the ionic current of the QL/AAO nanochannels was significantly increased, which should be attributed to the enhanced surface wettability of the nanochannel (Supplementary Fig. 26a, b). In addition, after the reaction, we observed a distinct red fluorescence by LSCM (Supplementary Fig. 26c). It's a similar finding to a reported 2022 study that claimed that the quinolinium skeleton in QL probes was involved in HSO_3^- attack to produce an additive complex, resulting in the ICT process and an apparent increase in fluorescence.[S2] The results demonstrated the successful construction of the QL/AAO nanosensor and the correctness of the device. More importantly, an increase in 2 V ionic current was registered even after nM-level SO_2 treatment (inset of Supplementary Fig. 26a), indicating that this QL/AAO nanochannel platform has a high sensitivity to SO_2.

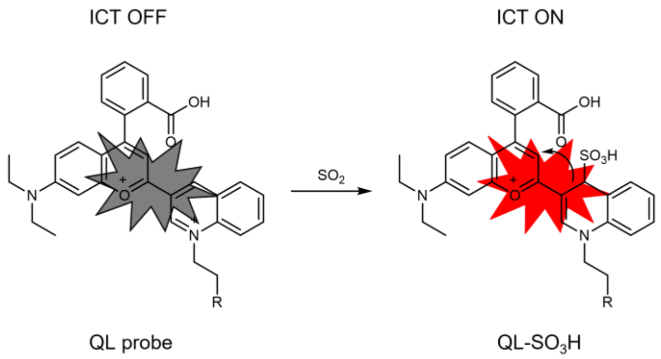

Supplementary Fig. 27 The intramolecular charge transfer (ICT) effect of the QL probe in response to SO_2.

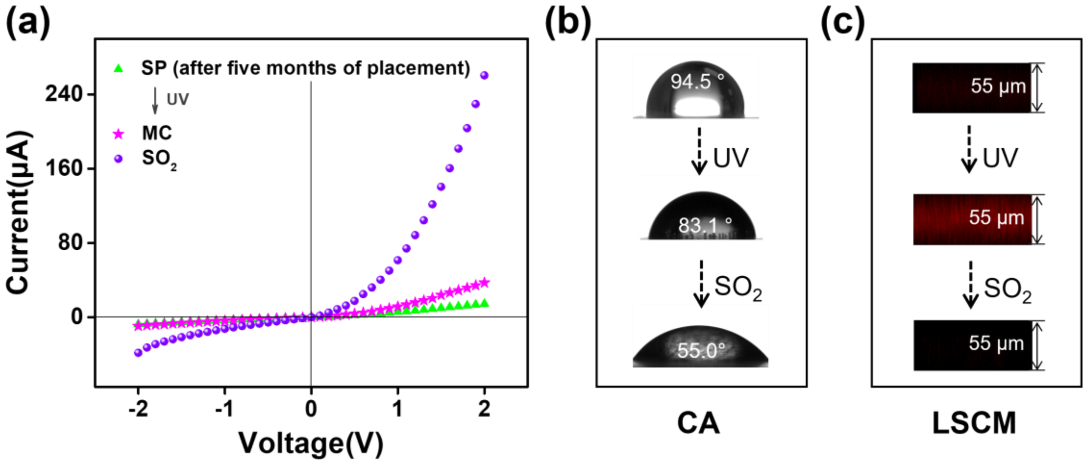

Supplementary Fig. 28 The response performance of the SP/AAO nanochannels to SO_2 after five months of placement.

As shown in Supplementary Fig. 28, after being placed for five months, our SP/AAO nanochannel still exhibited the desired light-operated SO_2 response performance. Under UV radiation, the transmembrane ionic current and surface hydrophilicity of the nanochannel was slightly increased. Red fluorescence was also observed due to photoisomerization. After the SO_2 response, the ionic current and hydrophilicity increased significantly. At this point, the red fluorescence again disappeared due to the disruption of the conjugate structure by nucleophilic addition. The response behaviour is the same as that of the freshly prepared SP/AAO nanosensor and the performance was not diminished. Therefore, our SP/AAO nanochannels have long-term chemical stability and can be stored for long periods of time.

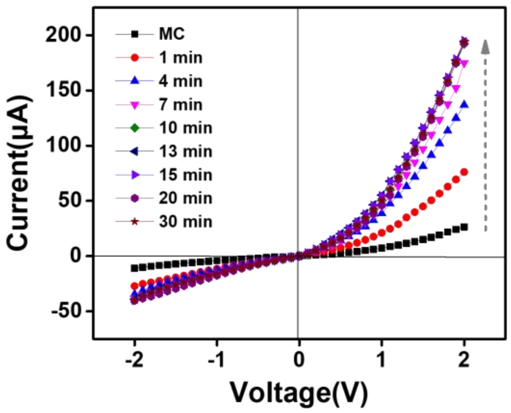

Supplementary Fig. 29 The change in I−V curve of the MC-state SP/AAO nanochannels with increasing SO_2 response time.

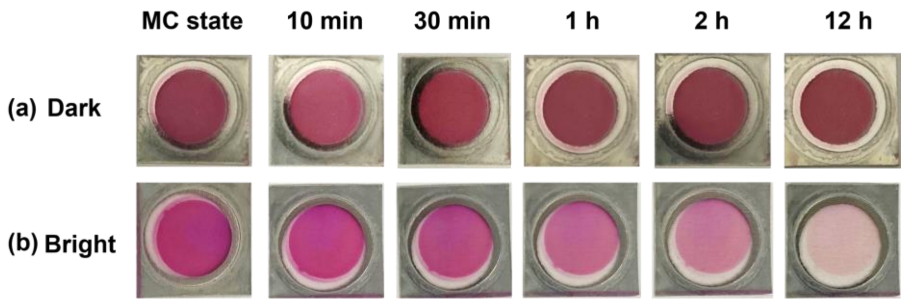

Supplementary Fig. 30 Colour change of MC-state SP/AAO nanochannels in (a) the darkroom and (b) a bright laboratory environment.

It has been reported that the closed-ring SP structure is stabilized if an electron-donating group such as methoxy is attached to the indoline ring; however, the opened-ring MC form is more stable if an electron-withdrawing group such as nitro or halogen is attached to the pyran ring.[S14,S15] Theoretically, our MC containing the -NO_2 substituent group is more stable. As shown in the optical photographs in Supplementary Fig. 30a, the MC-state SP/AAO membrane remained purple in a darkroom for 12 hours. Even when exposed to a bright laboratory environment (Supplementary Fig. 30b), it took 12 hours for the MC to fully return to the SP form. Therefore, the stability of MC is high, and almost no MC returned to SP within 13 min of SO_2 response.

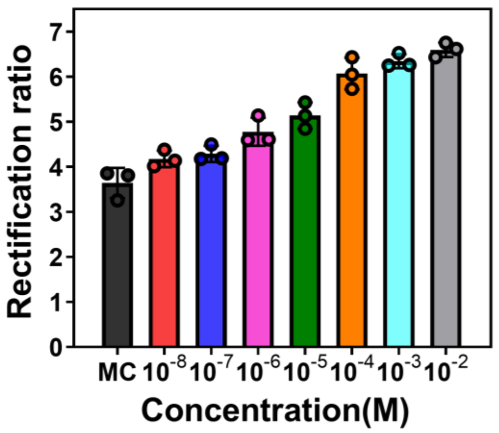

Supplementary Fig. 31 Rectification ratio changes of the MC-state SP/AAO nanochannels with increasing SO_2 concentration. Error bars denote the standard deviation from three different samples.

Supplementary Fig. 31 described the increasing trend of the rectification ratio as increased SO_2 concentration as a whole. The rectification ratio is defined as the ratio of the absolute values of the ionic currents at +2 V to the value at −2 V. As the SO_2 concentration gradually increases between 10^{-8} M and 10^{-2} M, the rectification ratio continued to increase, which should be related to the enhancement of wettability asymmetry on both sides of the nanochannels. Because the generation of more MC−SO_3H adducts would lead to a less hydrophobic nanochannel surface as SO_2 increases, while the wettability of the bottom surface remained hydrophobicity with CA ≈ 100°. As a result, the surface properties of the SP/AAO nanochannel membrane on both sides gradually varied from symmetric wettability to asymmetric wettability. As reported, this wettability asymmetry on both sides of the nanochannel will contribute to an improved rectification performance of the system.[S16] These corresponding statistic results about the rectification ratio have been shown in Supplementary Table 7.

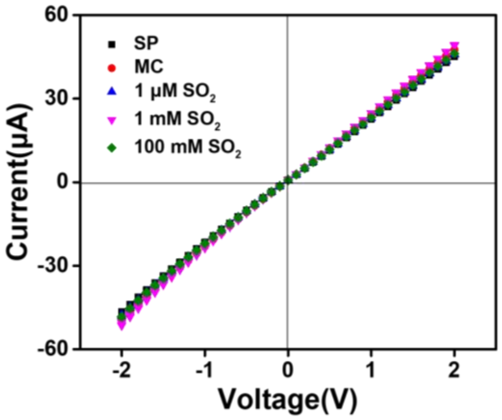

Supplementary Fig. 32 I−V curves of SP-modified cylindrical nanochannels before and after reacting with SO_2.

As a control experiment, cylindrical AAO nanochannels (pore diameter of 25 nm) were modified with SP to construct a symmetrical SP/AAO nanochannel system and further applied for SO_2 response in a light-controlled manner. As shown in Supplementary Fig. 32, the ionic current displayed a linear I−V curve, and the change in current is invisible. This phenomenon may be due to the symmetrical cylindrical geometry and lack of confinement effects from the barrier layer. Therefore, the asymmetric tubular nanochannels used in the system played an important role in the rectification performance and SO_2 response.

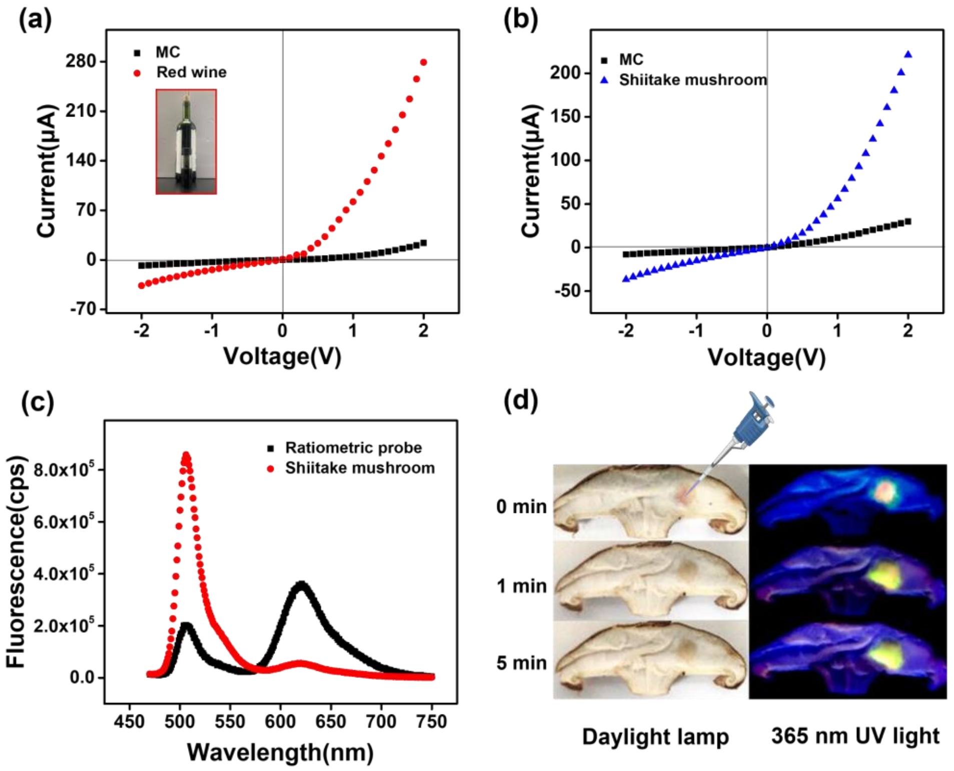

Supplementary Fig. 33 I−V curves of SP/AAO nanochannels before and after treatment with (a) red wine (inset shows the corresponding samples of red wine) or (b) shiitake mushroom. (c) Fluorescence changes of a reported ratiometric probe before and after treatment with shiitake mushroom. (d) Changes in optical and fluorescence on the mushroom sample when the probe was dropped onto a mushroom slice.

SO_2 is a permitted food additive, and it acts as preservatives and antioxidants in the food industry.[S17] However, excessive consumption would lead to damage to human health. Therefore, it is important to detect SO_2 in foods such as red wine and shiitake mushroom. In Supplementary Fig. 33a, we observed a significant increase in the ionic current of the nanochannels after red wine treatment, corresponding to an SO_2 content of about 1 mM in red wine. Upon exposure to a solution extracted from shiitake mushroom, the ionic current increased to 221 μA (Supplementary Fig. 33b), corresponding to an SO_2 content of ~35.6 mg kg^{-1}. In order to further confirm the qualification of the sensor, we used an already reported ratiometric probe to detect the same solution extracted from shiitake mushroom.[S18] As shown in Supplementary Fig. 33c, after treatment with the extracted solution, the probe's fluorescence peaked at 620 nm decreased while the emission at 505 nm increased, which proved the formation of the probe-SO_2 adduct. The fluorescence intensity ratio (I_{505}/I_{620}) demonstrated that the SO_2 content in the mushroom was 34.3 mg kg^{-1}, in general agreement with the detection results of nanochannels. Besides, we dropped the ratiometric probe onto the mushroom slices to directly display the presence of SO_2 in the mushroom. As shown in Supplementary Fig. 33d, red fluorescence can be observed when the probe has just touched the mushroom sample. However, after 1 min reaction, the red fluorescence of

the probe diminished significantly while the green fluorescence increased significantly. At 5 min, there is little red fluorescence was observed. Such a phenomenon allowed to confirm the presence of detectable SO_2 in the mushroom and to avoid false positives.

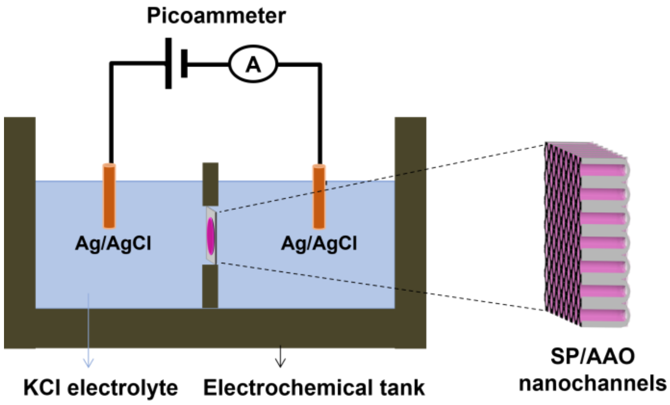

Supplementary Fig. 34 Schematic diagram of experimental apparatus for electrical measurement.

Supplementary Tables

Supplementary Table 1 Statistic results of current rectifying ratio in the process of fabricating SP/AAO nanochannels.

Rectifying ratio	AAO	APTES	SP-1h	SP-2h	SP-3h
Mean	3.45	5.31	3.94	1.89	1.68
SD	0.17	0.54	0.38	0.06	0.35

Supplementary Table 2 Elemental atomic ratios of SP/AAO nanochannels before and after reaction with SO_2.

Elemental atomic ratios (%)	C1s	N1s	O1s	Al2p	Si2p	S2p
SP/AAO	29.10	2.25	52.28	14.39	0.66	1.02
SO_2	31.45	2.90	48.72	13.87	1.22	1.83

Supplementary Table 3 Statistic results of current rectifying ratio during the UV-stimulated SO_2 response of SP/AAO nanochannels.

Rectifying ratio	SP	MC	SO_2
Mean	1.89	3.99	6.78
SD	0.06	0.06	0.52

Supplementary Table 4 Statistic results of I_{UV} of SP/AAO nanochannels.

I_{UV}(μA)	1 min	5 min	10 min	20 min
Mean	1.75	12.9	20.4	20.4
SD	0.88	0.69	1.42	0.20

Supplementary Table 5 Statistic results of I_{SO2} of SP/AAO nanochannels.

I_{SO2}(μA)	1 min	5 min	10 min	20 min
Mean	29.9	93.8	197	211
SD	1.28	1.02	6.54	1.99

Supplementary Table 6 Statistic results of ionic current of SP/AAO nanochannels with SO_2 response time.

Ionic current	MC	1 min	4 min	7 min	10 min	13 min	15 min	20 min	30 min
Mean	26.2	75.4	134.9	172.8	193.0	194.9	195.3	194.8	195.2
SD	0.35	0.88	2.30	2.44	2.08	0.61	0.30	1.66	1.05

Supplementary Table 7 Statistic results of current rectifying ratio of SP/AAO nanochannels with increasing SO_2 concentration.

Rectifying ratio	MC	10 nM	100 nM	1 μM	10 μM	100 μM	1 mM	10 mM
Mean	3.64	4.18	4.29	4.78	5.14	6.07	6.35	6.60
SD	0.33	0.19	0.18	0.31	0.29	0.35	0.16	0.16

Supplementary Table 8 Comparison of different sensors for SO_2 recognition through addition reactions.

SO_2 sensor	Signal	Linear range (μM)	Detection limit (μM)	Reference
Ly-NT-SP	Fluorescence	0-50	4.7	[S19]
SP-Gal	Fluorescence	0-50	0.04	[S20]
Benzo[e]indolium	Fluorescence	0-15	0.097	[S21]
Coumarin−hemicyanine	Fluorescence	0–15	0.38	[S22]
APPM-PET	Ionic current	1-5 000	1	[S13]
SP/AAO	Ionic current	0.01-1000	0.01	Our work

Supplementary References

[S1] Chen, L., Wu, J., Schmuck, C. & Tian, H. A switchable peptide sensor for real-time lysosomal tracking. *Chem. Commun.* **50**, 6443-6446 (2014).

[S2] Gong, W., Zhang, C., Zhang, X. & Shen, Y. Mitochondria-targetable colorimetric and far-red fluorescent sensor for rapid detection of SO_2 derivatives in food samples and living cells. Spectrochim. *Acta A Mol. Biomol. Spectrosc.* **278**, 121386 (2022).

[S3] Karnik, R., Castelino, K., Duan, C. & Majumdar, A. Diffusion-limited patterning of molecules in nanofluidic channels. *Nano Lett.* **6**, 1735-1740 (2006).

[S4] Zhang, D., Wang, Q., Fan, X., Zhang, M., Zhai, J. & Jiang L. An Effective Dark-Vis-UV Ternary Biomimetic Switching Based on N3/Spiropyran-Modified Nanochannels. *Adv. Mater.* **30**, 1804862 (2018).

[S5] Delamar, M., Albert, J. L., Aubard, J., & Dubois, J. E. XPS studies of photochromic molecules. II. Experimental polarity determination for merocyanines. J *Electron Spectros Relat Phenomena* **32**, 351-357 (1983).

[S6] Ivashenko, O., van Herpt, J. T., Feringa, B. L., Rudolf, P., & Browne, W. R. UV/vis and NIR light-responsive spiropyran self-assembled monolayers. *Langmuir* **29**, 4290-4297 (2013).

[S7] Ekiz, F., Oğuzkaya, F., Akin, M., Timur, S., Tanyeli, C., & Toppare, L. Synthesis and application of poly-SNS-anchored carboxylic acid: a novel functional matrix for biomolecule conjugation. *J. Mater. Chem. A* **21**, 12337-12343 (2011).

[S8] Dufrěne, Y. F., Vermeiren, H., Vanderleyden, J., & Rouxhet, P. G. Direct evidence for the involvement of extracellular proteins in the adhesion of Azospirillum brasilense. *Microbiology*, **142**, 855-865 (1996).

[S9] Lv, J., Xu, J., Zhao, X., Han, J., Chen, B., Wang, X., ... & Wang, L. Interface adhesion enhancement by condensed aromatic ring expansion of naphthalene imide derivatives for microvia metallization by copper electroplating. *Thin Solid Films*, **727**, 138671 (2021).

[S10] Genovese, M. E., Colusso, E., Colombo, M., Martucci, A., Athanassiou, A. & Fragouli, D. Acidochromic fibrous polymer composites for rapid gas detection. *J. Mater. Chem. A* **5**, 339-348 (2017).

[S11] Yang, K. et al. In Situ Laminated Separator Using Nitrogen–Sulfur Codoped Two-Dimensional Carbon Material to Anchor Polysulfides for High-Performance Li–S Batteries. *ACS Appl. Nano Mater.* **1**, 3807-3816 (2018).

[S12] Qiu, H., Ippolito, S., Galanti, A., Liu, Z., & Samori, P. Asymmetric dressing of WSe_2 with (macro) molecular switches: fabrication of quaternary-responsive transistors. *ACS nano* **15**, 10668-10677 (2021).

[S13] Chen, H., Xu, L., Tuo, W., Chen, X., Huang, J., Zhang, X. & Sun, Y. Fabrication of a smart nanofluidic biosensor through a reversible covalent bond strategy for high-efficiency bisulfite sensing and removal. *Anal. Chem.* **92**, 4131-4136 (2020).

[S14] Raymo, Françisco M., and Silvia Giordani. Signal processing at the molecular level. *J. Am. Chem. Soc.* **123**, 4651-4652 (2001).

[S15] Aldoshin, S. M., et al. Structures and photochromic properties of substituted spiroindolinonaphthoxazines. *Russ. Chem. Bull.* **47**, 1089-1097 (1998).

[S16] Zhang, Q., Hu, Z., Liu, Z., Zhai, J. & Jiang, L. Light-Gating Titania/Alumina

Heterogeneous Nanochannels with Regulatable Ion Rectification Characteristic. *Adv. Funct. Mater.* **24**, 424-431 (2014).

[S17] Gould, G. W. & Russell, N. J. Sulfite. *Food preservatives* 85-101(2003).

[S18] Lyu, J., Wang, C., & Zhang, X. Rational construction of a mitochondria-targeted reversible fluorescent probe with intramolecular fret for ratiometric monitoring sulfur dioxide and formaldehyde. *Biosensors* **12**, 715 (2022).

[S19] Zhang, W., Huo, F., Yue, Y., Zhang, Y., Chao, J., Cheng, F., & Yin, C. Heat stroke in cell tissues related to sulfur dioxide level is precisely monitored by light-controlled fluorescent probes. *J. Am. Chem. Soc.* **142**, 3262-3268 (2020).

[S20] Zhang, J., Fu, Y., Han, H. H., Zang, Y., Li, J., He, X. P., ... & Tian, H. Remote light-controlled intracellular target recognition by photochromic fluorescent glycoprobes. *Nat. Commun.* **8**, 987 (2017).

[S21] Sun, Y., Fan, S., Zhang, S., Zhao, D., Duan, L., & Li, R. A fluorescent turn-on probe based on benzo [e] indolium for bisulfite through 1, 4-addition reaction. *Sens. Actuators B Chem.* **193**, 173-177 (2014).

[S22] Sun, Y. Q., Liu, J., Zhang, J., Yang, T., & Guo, W. Fluorescent probe for biological gas SO_2 derivatives bisulfite and sulfite. *Chem. Commun.* **49**, 2637-2639 (2013).
